# Antidepressant use and dementia, cognitive measures, and neuroimaging outcomes: A population-based cohort study

**DOI:** 10.1017/S0033291726104942

**Published:** 2026-07-13

**Authors:** Xin Liu, Tengfei Lin, Yiwen Jiang, Shan Luo, Shanquan Chen, Yi Chai, Zhirong Yang, Ying Wei, Huali Wang, Xia Li, Yu-Tao Xiang, Feng Sha, Jinling Tang

**Affiliations:** 1 Unit of Psychiatry, Department of Public Health and Medicinal Administration, Faculty of Medicine, University of Macau, Macao SAR, China; 2 Department of Computational Biology and Medical Big Data, Shenzhen University of Advanced Technology, Shenzhen, Guangdong, China; 3 Shenzhen Institutes of Advanced Technology, Chinese Academy of Sciences, Shenzhen, Guangdong, China; 4 School of Pharmaceutical Sciences, Southern Medical University, Guangzhou, China; 5 Department of Family Medicine and Primary Care, School of Clinical Medicine, Li Ka Shing Faculty of Medicine, The University of Hong Kong, Hong Kong SAR, China; 6 School of Public Health, The University of Hong Kong, Hong Kong SAR, China; 7 School of Public Health, Shenzhen University Medical School, Shenzhen University, Shenzhen, Guangdong, China; 8 Department of Pharmacology and Pharmacy, LKS Faculty of Medicine, The University of Hong Kong, Hong Kong SAR, China; 9 The Hong Kong Jockey Club Centre for Suicide Research and Prevention, The University of Hong Kong, Hong Kong SAR, China; 10 Center for AI in Medicine, Artificial Intelligence Research Institute, University of Macau, Shenzhen, Guangdong, China; 11 Affiliated Shenzhen Maternity and Child Healthcare Hospital, Southern Medical University, Shenzhen 518048, Guangdong, China; 12 Dementia Care and Research Center, Clinical Research Division, Peking University Institute of Mental Health (Sixth Hospital), NHC Key Laboratory of Mental Health, National Clinical Research Center of Mental Disorders, Beijing, China; 13 Shanghai Mental Health Center, Shanghai Jiao Tong University School of Medicine, Shanghai, China

**Keywords:** antidepressant, cognitive function, dementia, neuroimaging

## Abstract

**Background:**

Prior observational studies have reported conflicting results regarding whether antidepressant treatment reduces long-term dementia risk, likely due to confounding by indication and reverse causation. We aimed to investigate the association between baseline antidepressant use and incident dementia, incorporating cognitive and neuroimaging outcomes.

**Methods:**

We conducted a prospective cohort study using UK Biobank participants free of dementia at baseline. Antidepressant use was self-reported at baseline (2006–2010). Incident dementia was identified through linked electronic health records until December 19, 2022. Cox proportional hazards models estimated hazard ratios (HRs) for all-cause dementia, Alzheimer’s disease (AD), and vascular dementia (VD), adjusting for sociodemographic, lifestyle, health-related, antidepressant indication factors, and co-medication of other anticholinergics. In subsamples, cognitive performance (*n* = 57,330) and structural brain imaging (*n* = 42,276) were examined as intermediate outcomes.

**Results:**

Among 461,464 participants, 33,721 (7.3%) reported baseline antidepressant use. Over a mean follow-up of 13.4 years, 7,922 (1.7%) developed incident dementia. Baseline antidepressant use was associated with higher risks of all-cause dementia (adjusted HR: 1.47, 95% CI 1.36–1.60), AD (1.53, 1.36–1.73), and VD (1.44, 1.23–1.70). Users performed worse on fluid intelligence and prospective memory tasks and showed lower total and gray matter volume, regional reductions in the hippocampal gray matter and basal nucleus, and greater white matter hyperintensity volume.

**Conclusions:**

Baseline antidepressant use was linked to a higher risk of dementia, poorer cognitive performance, and adverse brain structural changes. These findings underscore the importance of judicious prescribing, regular cognitive monitoring, and consideration of non-pharmacological approaches in clinical care.

## Introduction

Dementia, a progressive neurodegenerative disorder affecting 57.4 million people worldwide in 2019 and projected to reach 152.8 million by 2050, presents significant challenges and underscores the need for scalable prevention strategies (Nichols et al., 2022). With no curative treatments available, prioritizing the management of modifiable risk factors has become crucial in dementia prevention. Depression, particularly in midlife, is recognized as a key modifiable risk factor for dementia, with well-established links to cognitive decline and neurodegeneration (Li et al., 2025; Livingston et al., 2024). Consequently, it has been hypothesized that effective treatment of depression could reduce the risk of developing dementia. Antidepressants are widely prescribed not only for depression, but also for other conditions such as anxiety, chronic pain, and insomnia (Cipriani et al., 2018; Olfson, Blanco, & Marcus, 2016; Tamblyn et al., 2019). These conditions are also associated with an elevated dementia risk, suggesting that antidepressant use may influence dementia outcomes through multiple pathways.

Evidence on the association between antidepressant use and dementia risk remains inconsistent. While meta-analysis of longitudinal studies concluded that antidepressant use was associated with increased dementia risk among individuals with depression (relative risk, RR: 1.37, 95% CI 1.11–1.70) (Lin et al., 2020), other studies suggested protective effects (Yang et al., 2023) or no significant association (Ramos-Cejudo et al., 2024; Su et al., 2020; vom Hofe et al., 2024). A major challenge in interpreting this literature is confounding by indication, because antidepressants are prescribed not only for depression, an established risk factor for dementia, but also for anxiety, insomnia, and chronic pain, which may themselves be associated with dementia risk (Kuring, Mathias, & Ward, 2020; Shi et al., 2018; Whitlock et al., 2017). Reverse causation is also difficult to exclude, as antidepressants may be prescribed for early neuropsychiatric manifestations of dementia before formal diagnosis (Neațu et al., 2024). Accordingly, in the present study, we sought to address these challenges by jointly accounting for depression diagnosis, depressive symptom severity, and other common antidepressant indications, while incorporating cognitive and neuroimaging measures to better characterize potential preclinical changes.

A recent analysis from the Rotterdam Study (vom Hofe et al., 2024) represents one of the most methodologically rigorous attempts to address these limitations. It simultaneously adjusted for depression diagnosis and symptom severity, while incorporating cognitive assessments and structural brain magnetic resonance imaging (MRI) to help mitigate reverse causation. Notably, the study observed a positive association between antidepressant use and incident dementia, although the results did not reach statistical significance. This is likely due to the modest sample size (*N* ≈ 5,500) and a relatively small number of dementia events (<700), which limits the statistical power to detect modest associations with confidence. These findings underscore the need for larger prospective studies that can extend this approach and evaluate the association more precisely.

To this end, we conducted a study using data from the UK Biobank, a large, population-based cohort with long-term follow-up and comprehensive clinical, cognitive, and neuroimaging data. We aimed to examine the association between baseline antidepressant use and the risk of incident dementia, and to assess whether this association persists across indication-defined subgroups, thereby addressing confounding by indication. We further incorporated cognitive test performance and structural brain imaging as complementary markers to better characterize potential preclinical cognitive and neurobiological correlates and to help mitigate concerns about reverse causation. By integrating multidimensional data from a well-powered cohort, this study aims to provide epidemiological and neurostructural evidence to better characterize the association between antidepressant use and cognitive health.

## Methods

### Study design

This prospective cohort study was based on data from the UK Biobank, a large-scale prospective cohort of over 500,000 adults aged 37 to 73 years at recruitment between 2006 and 2010 across 22 centers in England, Scotland, and Wales. Baseline assessments included detailed information on medication use, sociodemographic factors, lifestyle behaviors, and health indicators collected via touchscreen questionnaires, interviews, and physical measurements. Cognitive assessments were available at baseline (2006–2010) and during imaging visits beginning in 2014, whereas structural neuroimaging data were available only during imaging visits (2014+). Incident dementia diagnoses were prospectively ascertained through linkage to electronic health records (EHRs), including primary care, hospital inpatient admissions, death registry, and cancer registry data. Ethical approval was granted by the UK Biobank Research Ethics Committee (REC reference: 11/NW/0382).

### Participants

Participants were excluded if they had a diagnosis of dementia at baseline or were missing data on one or more key covariates. All participants provided touchscreen informed consent.

### Antidepressant exposure

The primary exposure was self-reported baseline antidepressant use, assessed using touchscreen questionnaires and verbal interviews (Supplementary Table 1). Participants were asked to report their regular prescription medications, defined as treatments taken on most days of the week during the past 4 weeks (Lin et al., 2025; Yang et al., 2023). At baseline, only prescription medications that were actually taken regularly were recorded, while short-term treatments (e.g., a 1-week course of antibiotics for an infection) and medications that had been recently discontinued were excluded. Medication entries were mapped to UK Biobank Field 20003, which comprises 6,745 codes. Following established methods (Wu et al., 2019), categories with at least 10 users were retained, and active ingredients were classified according to the Anatomical Therapeutic Chemical (ATC) system, with antidepressants identified under ATC code N06A (Supplementary Table 2).

Given the potential cognitive risks associated with anticholinergic drugs and the varying mechanisms of action across antidepressant subtypes, secondary exposures included the anticholinergic burden and pharmacological subtypes of antidepressants (Harmer, Duman, & Cowen, 2017; Richardson et al., 2018). Due to the lack of consensus on the classification of drugs’ anticholinergic activity, we adopted three commonly used criteria: (1) the integrated approach proposed by Coupland et al. (Coupland et al., 2019), (2) the 2012 updated Anticholinergic Cognitive Burden (ACB) scale (Richardson et al., 2018), and (3) the pharmacology-based classification (Lavrador et al., 2023). To improve the robustness, each criterion was applied separately, and the corresponding classification of antidepressants under each criterion is presented in Supplementary Table 3. Pharmacological subtypes were defined as tricyclic antidepressants (TCAs), selective serotonin reuptake inhibitors (SSRIs), serotonin–norepinephrine reuptake inhibitors (SNRIs), noradrenergic and specific serotonergic antidepressants (NaSSAs), and others.

### Dementia outcome

Incident dementia diagnoses were identified through UK Biobank’s algorithmically derived first occurrence fields, integrating data from linked self-report, hospital, primary care, and death record data (Supplementary Table 1). Diagnoses were defined according to ICD-10 codes: all-cause dementia (F00–F03, G30), Alzheimer’s disease (AD: F00, G30), and vascular dementia (VD: F01). The diagnosis date was taken as the earliest recorded entry across any data source. Participants were followed from baseline until the earliest of dementia diagnosis, death, or the censoring date (December 19, 2022). Validation studies have reported positive predictive values of 82.5% for all-cause dementia and lower for subtypes (AD: 71.4%, VD: 43.8%) (Wilkinson et al., 2019).

### Cognitive outcome

Cognitive function was assessed at both baseline (2006–2010) and during the imaging visit (2014+), using two computerized tests administered via touchscreen interface. Fluid intelligence, reflecting reasoning and problem-solving ability independent of prior learning, was measured using a 13-item task with a score range of 0 to 13, based on the number of correct responses within a 2-minute limit. Episodic memory was assessed using the Prospective Memory Test, in which participants were instructed to perform a task later in the session; performance was dichotomized as correct on first attempt versus incorrect. Specific field identifiers are provided in Supplementary Table 1. In the present analysis, cognitive outcomes were based on measures obtained at the imaging visit (2014+), while baseline cognitive measures were used in sensitivity analyses.

### Neuroimaging outcomes

Structural brain imaging was obtained during UK Biobank imaging visits (2014+) starting in 2014 and was conducted using 3T MRI scanners under standardized protocols developed by the UK Biobank imaging team (Alfaro-Almagro et al., 2018; Miller et al., 2016). T1-weighted and T2-weighted fluid-attenuated inversion recovery (FLAIR) sequences were used to derive volumetric measures of brain structure.

The neuroimaging outcomes of interest were brain areas most likely reflecting the cognitive function and antidepressant effects reported in previous studies. These included total brain volume as an indicator of global brain size (Bigler & Tate, 2001); gray and white matter volumes, reflecting different parts of neurons that include cell bodies, dendrites, synapses, and axons for gray matter and (un)myelinated axons for white matter(Purves et al., 2025); hippocampal volume and hippocampal gray matter volume, indexing learning and memory(Rao et al., 2022); cerebellum-cortex volume, reflecting cognitive–motor integration(Koziol et al., 2014); lateral orbitofrontal cortex volume, involved in non-reward processing and emotion regulation(B. Zhang et al., 2024); basal nucleus volume, related to emotional, motivational, associative, and cognitive functions (Geula et al., 2021; Herrero, Barcia, & Navarro, 2002); and white matter hyperintensities, as markers of cerebrovascular pathology (Gaubert et al., 2021). Specific field identifiers and calculation methods are provided in Supplementary Table 1.

### Covariates

Potential confounders available in the UK Biobank were chosen based on clinical insight and previous reviews (Livingston et al., 2024), including sociodemographic, lifestyle, health-related, and antidepressant indication factors, as well as the comedication of other anticholinergics. Sociodemographic variables included age, sex, ethnicity (White vs other), less education (less than university degree), living alone, and socioeconomic status (Townsend deprivation index: low, intermediate, high). Lifestyle behaviors comprised current smoking, excessive alcohol consumption (>21 units/week), physical inactivity, sleep duration (<7, 7–8, ≥8 hours/day), and social isolation. Health indicators included self-rated overall health, hypertension, diabetes, hyperlipidemia, traumatic brain injury, hearing loss, vision loss, and obesity (BMI ≥30 kg/m^2^). To address confounding by indication, we additionally adjusted for depression, the Patient Health Questionnaire-2 (PHQ-2) total score (a two-item measure of depressive symptom severity over the past 2 weeks), anxiety disorders, chronic pain, and insomnia (self-reported sleep problems). Finally, concomitant use of other anticholinergic medications (non-antidepressants) was considered. A pharmacology-based classification of anticholinergics (Lavrador et al., 2023) was applied to comprehensively account for anticholinergic comedication (Supplementary Table 2). Detailed descriptions of covariates are provided in Supplementary Table 1.

### Statistical analysis

Baseline characteristics were summarized as means (SD) or percentages and compared using standardized mean differences (SMDs), with SMD >0.1 considered a meaningful imbalance.

The association between baseline antidepressant use and incident all-cause dementia was pre-specified as the primary analysis. AD and VD were examined as secondary subtype outcomes. Analyses of cognitive performance and neuroimaging markers were pre-specified intermediate outcomes. Analyses stratified by clinical indications, baseline demographic subgroups, antidepressant pharmacological subclasses, and anticholinergic burden were exploratory and interpreted as hypothesis-generating.

Cox proportional hazards models were used to estimate hazard ratios (HRs) and 95% confidence intervals (CIs) for the association between baseline antidepressant use and incident dementia (all-cause dementia, AD, and VD individually). To account for potential confounding and to illustrate the influence of different covariate domains on the association between antidepressant use and dementia risk, a series of models was fitted sequentially. Model 1 adjusted for basic sociodemographic factors (age, sex, ethnicity, socioeconomic status, less education, and living alone status); Model 2 additionally adjusted for lifestyle variables (excessive alcohol consumption, current smoking status, physical inactivity, sleeping duration categories, and social isolation); Model 3 further included health indicators (overall health, hyperlipidemia, diabetes, hypertension, traumatic brain injury, hearing loss, vision loss, and obesity); Model 4 incorporated antidepressant indication–related variables (depression, PHQ-2 total score, anxiety, self-reported chronic pain, and self-reported insomnia); Model 5 additionally adjusted for using other anticholinergics.

Stratified analyses were conducted to explore potential effect heterogeneity across indication and demographic subgroups. Specifically, stratification by antidepressant indications was performed to examine whether the observed associations persisted among individuals with and without underlying conditions warranting antidepressant use. This approach aimed to evaluate the consistency of the association irrespective of antidepressant-relevant indications, thereby minimizing concerns of confounding by indication. In addition, stratification by demographic characteristics was performed to identify potentially sensitive populations in whom the association between antidepressant use and dementia risk might differ in magnitude. To formally assess effect modification, multiplicative interaction terms between antidepressant use and each demographic variable were tested within fully adjusted models. Within each stratum, HRs were estimated using fully adjusted models, excluding the stratified variable from the adjustment set to avoid overadjustment.

To examine the association between baseline antidepressant use and cognitive outcomes, multiple linear regression models were applied for fluid intelligence, and multiple logistic regression was used for prospective memory, which was treated as a binary variable. The same modeling sequence (Models 1 to 5) was used for adjustment.

Multiple linear regression models were conducted to assess the association between baseline antidepressant use and neuroimaging outcomes. To reduce inter-individual variability, all neuroimaging volume measures were normalized for head size using the scaling factors provided by the UK Biobank. For the primary analyses, standardized *z*-scores were derived for each outcome. Sensitivity analyses were conducted using raw volumes and unnormalized measures to assess robustness. Because nine neuroimaging outcomes were examined in the primary neuroimaging analysis, Bonferroni correction was applied to the fully adjusted model (Model 5), with statistical significance defined as *p* < 0.05/9 = 0.0056.

For both cognitive and neuroimaging outcomes, the magnitude of the association with baseline antidepressant use was compared with that of a 1-year increase in age at baseline, based on estimates from Model 5 (Lee et al., 2008).

Exploratory analyses assessed dementia risk associated with antidepressants classified by anticholinergic burden and pharmacological subtype, using non-users as the reference group. HRs were estimated using the same sequence of adjustment models.

**Figure 1.**
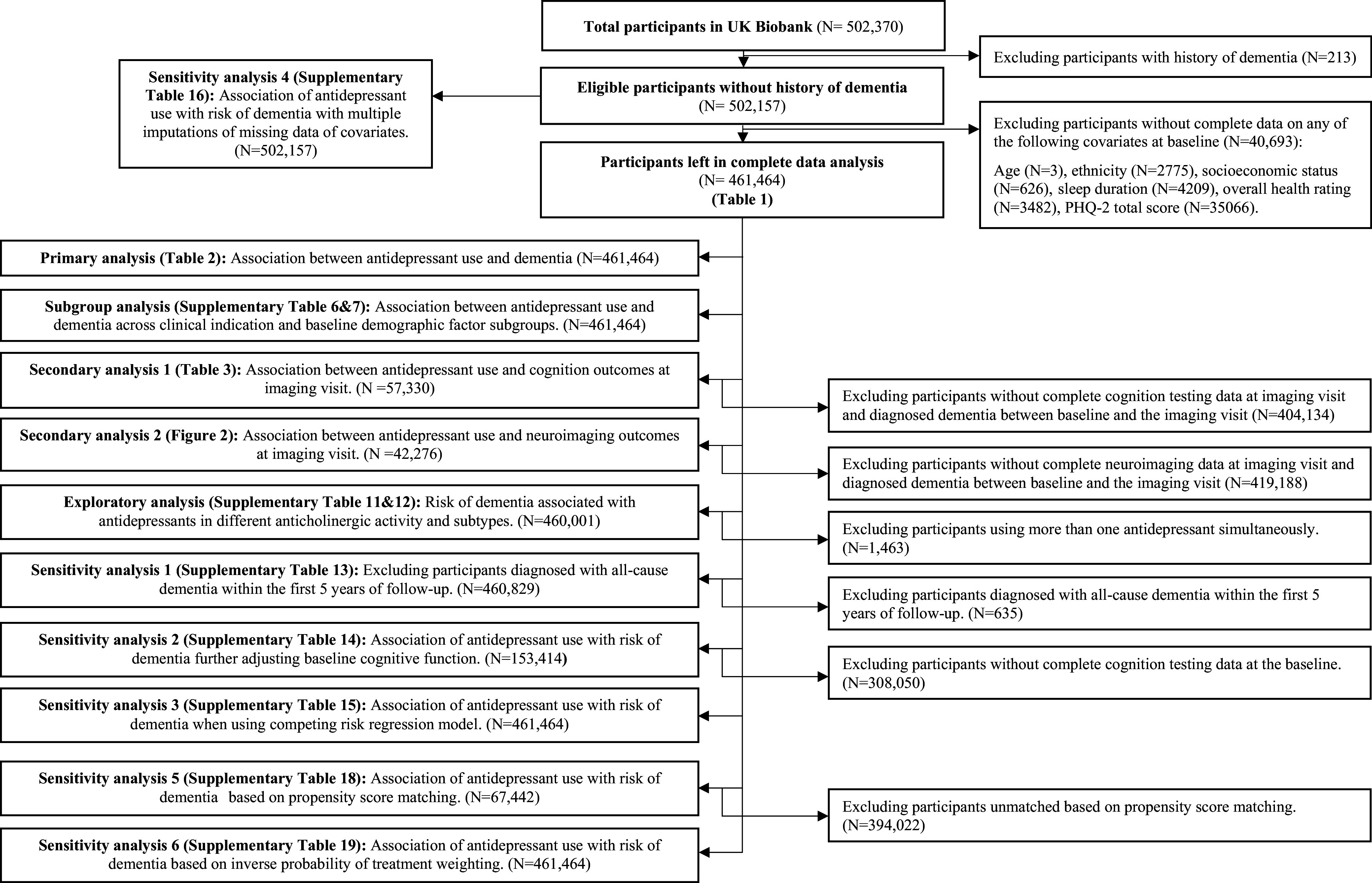
Flow diagram of study participants. Figure 1. long description.

Sensitivity analyses were conducted to evaluate the robustness of our findings. First, to minimize the potential influence of reverse causation, we repeated the primary analysis after excluding participants who developed dementia within the first 5 years of follow-up. Second, given the potential role of baseline cognitive performance in mitigating reverse causation, we repeated the primary analysis with additional adjustment for baseline cognitive test scores (fluid intelligence and prospective memory), to assess whether the observed associations were independent of initial cognitive status. Third, to account for the competing risk of death, we applied competing risks regression models, treating death as a competing event. Fourth, to address the potential bias from missing data, we performed multiple imputations using chained equations for all covariates included in the fully adjusted model. A total of 15 imputed datasets were generated, as there were 8.1% (40,693/502,157) of participants having a missing value for at least one of the variables (Figure 1). Variables included in the imputation model were dementia status, follow-up time, and all covariates from the fully adjusted model. Fifth, to further mitigate confounding by indication, we applied propensity score matching (PSM) to create a matched cohort of baseline antidepressant users and non-users based on similar propensity scores. These scores were estimated using a logistic regression model, considering baseline antidepressant use and the previously mentioned covariates. A 1:1 matching protocol was used with nearest-neighbor matching without replacement. SMDs were calculated to assess the balance between matched groups. Fully adjusted Cox proportional hazards models were then used to estimate HRs and 95% CIs for the association between baseline antidepressant use and incident dementia (all-cause dementia, AD, and VD individually) in the matched cohort. Sixth, to further adjust for potential confounders, we applied inverse probability of treatment weighting (IPTW), weighting participants by the inverse of their estimated probability of receiving antidepressant treatment. To address potential issues with extreme weights, we applied weight truncation, capping weights at the 1st and 99th percentiles. Weighted Cox proportional hazards models were used to estimate HRs and 95% CIs for the association between baseline antidepressant use and incident dementia with full adjustment. Significance level was set at 0.05 (two-tailed) unless otherwise specified.

## Results

### Baseline characteristics

Among 502,157 eligible participants, 461,464 with complete baseline data were included in the analysis (Figure 1). The baseline characteristics of eligible and included participants were similar (Supplementary Table 4). Among those with complete baseline data, the mean age was 57 years (SD 8.1), 45.8% were male, and 95.2% were White (Table 1). At baseline, 33,721 participants (7.3%) reported antidepressant use. Compared with non-users, baseline antidepressant users were more likely to be female, have less education, live alone, and report poorer self-rated overall health. They also exhibited higher prevalence of unhealthy lifestyle factors such as smoking, physical inactivity, and suboptimal sleep duration, as well as social isolation, but a lower prevalence of excessive alcohol consumption. Socioeconomic status differed between groups, with users more likely to be in the high deprivation category. Antidepressant users had a greater comorbidity burden, including obesity, diabetes, hyperlipidemia, and hearing loss, and were more likely to report depression, anxiety, chronic pain, and insomnia. The mean PHQ-2 score was substantially higher among users (1.54 vs 0.50), and they had higher use of other anticholinergics.

**Table 1.** Baseline characteristics of participants Table 1. long description.

Variable	All participants	Non-antidepressant use	Antidepressant use	SMD
*N* (%)	461,464	427,743 (92.7)	33,721 (7.3)	
Age, years	57 (8.1)	57 (8.1)	56 (7.8)	0.015
Male	211,520 (45.8)	201,041 (47.0)	10,479 (31.1)	**0.331**
White	439,517 (95.2)	406,818 (95.1)	32,699 (97.0)	0.096
Socioeconomic status				**0.172**
Low	95,466 (20.7)	89,644 (21.0)	5,822 (17.3)	
Intermediate	278,107 (60.3)	258,734 (60.5)	19,373 (57.5)	
High	87,891 (19.0)	79,365 (18.6)	8,526 (25.3)	
Less education	281,077 (60.9)	257,875 (60.3)	23,202 (68.8)	**0.179**
Living alone	84,344 (18.3)	75,906 (17.7)	8,438 (25.0)	**0.178**
Excessive alcohol consumption	107,817 (23.4)	101,845 (23.8)	5,972 (17.7)	**0.151**
Current smoking	47,560 (10.3)	42,103 (9.8)	5,457 (16.2)	**0.189**
Physical inactivity	71,015 (15.4)	63,687 (14.9)	7,328 (21.7)	**0.178**
Sleep duration, hour				**0.250**
<7	111,953 (24.3)	103,047 (24.1)	8,906 (26.4)	
7–8	179,608 (38.9)	170,077 (39.8)	9,531 (28.3)	
> = 8	169,903 (36.8)	154,619 (36.1)	15,284 (45.3)	
Social isolation	64,856 (14.1)	58,537 (13.7)	6,319 (18.7)	**0.137**
Overall health				**0.735**
Poor	19,739 (4.3)	14,011 (3.3)	5,728 (17.0)	
Fair	93,095 (20.2)	81,241 (19.0)	11,854 (35.2)	
Good	269,621 (58.4)	255,306 (59.7)	14,315 (42.5)	
Excellent	79,009 (17.1)	77,185 (18.0)	1,824 (5.4)	
Hyperlipidemia	67,876 (14.7)	60,952 (14.2)	6,924 (20.5)	**0.166**
Diabetes	133,823 (29.0)	121,258 (28.3)	12,565 (37.3)	**0.191**
Hypertension	257,119 (55.7)	237,676 (55.6)	19,443 (57.7)	0.042
Traumatic brain injury	6,987 (1.5)	6,123 (1.4)	864 (2.6)	0.081
Hearing loss	123,309 (26.7)	112,361 (26.3)	10,948 (32.5)	**0.136**
Vision loss	39,188 (8.5)	35,902 (8.4)	3,286 (9.7)	0.047
Obesity	110,699 (24.0)	99,180 (23.2)	11,519 (34.2)	**0.244**
Depression	37,575 (8.1)	18,071 (4.2)	19,504 (57.8)	**1.422**
PHQ-2 total score	0.58 (1.11)	0.50 (1.01)	1.54 (1.71)	**0.740**
Anxiety	24,820 (5.4)	17,145 (4.0)	7,675 (22.8)	**0.573**
Chronic pain	197,938 (42.9)	175,875 (41.1)	22,063 (65.4)	**0.502**
Insomnia	127,509 (27.6)	113,134 (26.4)	14,375 (42.6)	**0.345**
Using other Anticholinergics	22,584 (4.9)	17,187 (4.0)	5,397 (16.0)	**0.408**

*Note*: Bolded values: SMD > 0.1.

PHQ-2, Patient Health Questionnaire-2.

### Association between baseline antidepressant use and incident dementia

Among the 461,464 participants, a total of 7,922 all-cause dementia cases, 3,777 AD cases, and 1,859 VD cases were recorded over a mean follow-up of 13.4 (2.1) years (Supplementary Table 5). Specifically, 1,018 (3.0%) baseline antidepressant users and 6,904 (1.6%) non-users developed dementia (Table 2). In Model 1, baseline antidepressant use was associated with over twofold increased risk of all-cause dementia (HR 2.13, 95% CI 1.99–2.28), adjusting for baseline demographic factors. This association remained significant after full adjustment for sociodemographic, lifestyle, health, antidepressant indication variables, and co-medication of other anticholinergics (adjusted HR 1.47, 95% CI 1.36–1.60). Associations were also observed for AD (adjusted HR 1.53, 1.36–1.73) and VD (adjusted HR 1.44, 1.23–1.70).

**Table 2.** Association of antidepressant use with risk of dementia Table 2. long description.

	Non-antidepressant use	Antidepressant use	*p*
*N*	427,743	33,721	
**All-cause dementia**			
Number (%) of dementia events	6,904 (1.6)	1,018 (3.0)	
HR (95% CI)			
Model 1^a^	Ref	**2.13 [1.99, 2.28]**	**<0.001**
Model 2^b^	Ref	**2.07 [1.94, 2.21]**	**<0.001**
Model 3^c^	Ref	**1.67 [1.56, 1.79]**	**<0.001**
Model 4^d^	Ref	**1.50 [1.39, 1.63]**	**<0.001**
Model 5^e^	Ref	**1.47 [1.36, 1.60]**	**<0.001**
**Alzheimer’s disease**			
Number (%) of Alzheimer’s disease event	3,320 (0.8)	457 (1.4)	
HR (95% CI)			
Model 1^a^	Ref	**1.93 [1.75, 2.13]**	**<0.001**
Model 2^b^	Ref	**1.92 [1.74, 2.12]**	**<0.001**
Model 3^c^	Ref	**1.68 [1.52, 1.86]**	**<0.001**
Model 4^d^	Ref	**1.56 [1.38, 1.76]**	**<0.001**
Model 5^e^	Ref	**1.53 [1.36, 1.73]**	**<0.001**
**Vascular dementia**			
Number (%) of vascular dementia events	1,584 (0.4)	275 (0.8)	
HR (95% CI)			
Model 1^a^	Ref	**2.57 [2.25, 2.92]**	**<0.001**
Model 2^b^	Ref	**2.44 [2.14, 2.78]**	**<0.001**
Model 3^c^	Ref	**1.79 [1.57, 2.05]**	**<0.001**
Model 4^d^	Ref	**1.49 [1.27, 1.75]**	**<0.001**
Model 5^e^	Ref	**1.44 [1.23, 1.70]**	**<0.001**

*Note*: Bolded values: *p* < 0.05.

a Model 1: Baseline demographic factors: age, sex, ethnicity, socioeconomic status, less education, and living alone status.

b Model 2: Model 1 + lifestyle factors: excessive alcohol consumption, current smoking status, physical inactivity, sleeping duration categories, and social isolation.

c Model 3: Model 2 + health indicators: overall health, hyperlipidemia, diabetes, hypertension, traumatic brain injury, hearing loss, vision loss, and obesity.

d Model 4: Model 3 + antidepressant indication factors: depression, PHQ-2 total score, anxiety, self-reported chronic pain, and self-reported insomnia.

e Model 5: Model 4 + using other anticholinergics.

### Stratified analyses by antidepressant indication

Stratified analyses showed that the association between baseline antidepressant use and dementia remained significant regardless of underlying antidepressant indications (Supplementary Table 6). Adjusted HRs ranged from 1.31 (95% CI 1.15–1.50) among individuals with insomnia to 1.62 (1.48–1.77) among those with PHQ-2 scores ≤2.

### Subgroup analyses by baseline demographics

In subgroup analyses, baseline antidepressant use was consistently associated with increased risk of dementia across most demographic strata, except for ‘Others’ in ethnicity (Supplementary Table 7). Interaction effects were significant for age (*p* = 0.002), education (*p* = 0.008), and living alone (*p* = 0.013). The adjusted HRs were higher among those aged <60 years (1.49, 95% CI 1.19–1.86), those with higher education (1.67, 1.41–1.97), and participants not living alone (1.54, 1.40–1.69).

### Association with cognitive outcomes

Among 57,330 participants with cognitive testing data, baseline antidepressant use was associated with lower fluid intelligence (adjusted *β* −0.17, 95% CI −0.25 to −0.08) and reduced odds of correct prospective memory performance (adjusted OR 0.81, 0.73 to 0.91). These effects were equivalent to an increase of 7.3 and 3.9 years of age at baseline, respectively (Table 3).

**Table 3.** Association between antidepressant use and cognitive function (fluid intelligence and prospective memory) at imaging visit Table 3. long description.

Cognitive function outcome	Non-antidepressant use	Antidepressant use	*p*
**Number of participants**	54,433	2,897	
**Fluid intelligence score**			
Mean (SD)	6.56 (2.06)	6.27 (2.03)	
*β* (95% CI)			
Model 1^a^	Ref	**−0.20 [−0.28, −0.13]**	**<0.001**
Model 2^b^	Ref	**−0.19 [−0.26, −0.12]**	**<0.001**
Model 3^c^	Ref	**−0.11 [−0.18, −0.03]**	**0.004**
Model 4^d^	Ref	**−0.16 [−0.25, −0.08]**	**<0.001**
Model 5^e^	Ref	**−0.17 [−0.25, −0.08]**	**<0.001**
**Prospective memory result**			
Number (%) of positive prospective memory	44,998 (82.7)	2,311 (79.8)	
OR (95% CI)			
Model 1^a^	Ref	**0.80 [0.73, 0.88]**	**<0.001**
Model 2^b^	Ref	**0.80 [0.73, 0.88]**	**<0.001**
Model 3^c^	Ref	**0.84 [0.76, 0.92]**	**<0.001**
Model 4^d^	Ref	**0.81 [0.72, 0.90]**	**<0.001**
Model 5^e^	Ref	**0.81 [0.73, 0.91]**	**<0.001**

*Note*: Bolded values: *p* < 0.05.

a Model 1: Baseline demographic factors: age, sex, ethnicity, socioeconomic status, less education, and living alone status.

b Model 2:    Model 1 + lifestyle factors: excessive alcohol consumption, current smoking status, physical inactivity, sleeping duration categories, and social isolation.

c Model 3:    Model 2 + health indicators: overall health, hyperlipidemia, diabetes, hypertension, traumatic brain injury, hearing loss, vision loss, and obesity.

d Model 4:    Model 3 + antidepressant indication factors: depression, PHQ-2 total score, anxiety, self-reported chronic pain, and self-reported insomnia.

e Model 5:    Model 4 + using other anticholinergics.

### Association with neuroimaging markers

**Figure 2.**
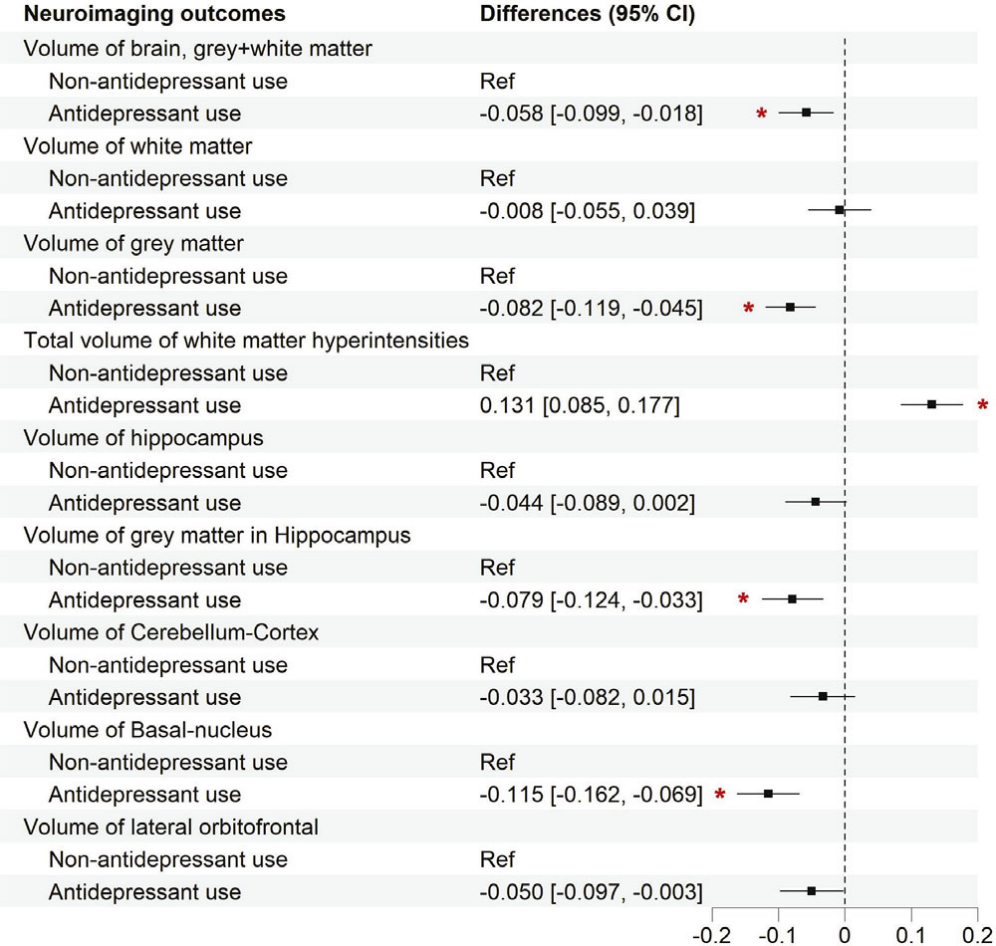
Forest plot of the association between antidepressant use and neuroimaging outcomes (*z*-score). *Note:* ^a^All neuroimaging outcomes for each individual were normalized by head size and converted to *z*-scores based on the mean and standard deviation. ^b^Model was adjusted for age, sex, ethnicity, socioeconomic status, less education, living alone status, excessive alcohol consumption, current smoking status, physical inactivity, sleeping duration categories, social isolation, overall health, hyperlipidemia, diabetes, hypertension, traumatic brain injury, hearing loss, vision loss, obesity, depression, PHQ-2 total score, anxiety, self-reported chronic pain, self-reported insomnia, and using other anticholinergics. *Associations that remained significant after Bonferroni correction across the nine neuroimaging outcomes. Figure 2. long description.

Neuroimaging data were available for 42,276 participants. In fully adjusted models, baseline antidepressant use was associated with lower total brain volume (*β* −0.058, 95% CI −0.099 to −0.018) and gray matter volume (*β* −0.082, −0.119 to −0.045), equivalent to an increase of 0.8 and 1.1 years of age at baseline, respectively. Regional reductions were observed in the gray matter of the hippocampus (*β* −0.079, −0.124 to −0.033) and basal nucleus (*β* −0.115, −0.162 to −0.069), equivalent to an increase of 2.1 and 2.5 years of age at baseline, respectively. Baseline antidepressant use was also associated with a higher volume of white matter hyperintensities ( *β* 0.131, 0.085 to 0.177), equivalent to 3.0 years of age. These associations remained significant after Bonferroni correction across the nine neuroimaging outcomes. An inverse association with lateral orbitofrontal cortex volume was also observed (*β* −0.050, −0.097 to −0.003; equivalent to 1.3 years of older age at baseline), but this did not remain significant after Bonferroni correction (Figure 2, Supplementary Table 8).

The associations were directionally consistent when using head-size-normalized volumetric measures (Supplementary Table 9). In fully adjusted models, baseline antidepressant use was associated with lower total and gray matter volumes, regional reductions in hippocampal gray matter, the basal nucleus, and the lateral orbitofrontal cortex, and a greater white matter hyperintensity volume. In analyses using crude volumetric measures (Supplementary Table 10), the associations were generally attenuated after full adjustment, but remained evident for white matter hyperintensity volume and basal nucleus volume, providing supportive evidence for the primary findings.

### Anticholinergic burden and antidepressant subtype

Across various anticholinergic criteria, analyses of anticholinergic burden showed elevated dementia risk for both non-anticholinergic (adjusted HR range from 1.52–1.63) and anticholinergic antidepressants (adjusted HR range from 1.33 to 1.46) (Supplementary Table 11). By pharmacological class, the association remained significant for SSRIs (adjusted HR 1.60, 1.43–1.80), TCAs (adjusted HR 1.31, 1.17–1.47), SNRIs (adjusted HR 1.67, 1.30–2.15), and NaSSAs (adjusted HR 2.05, 1.52–2.79), but not for other agents (adjusted HR 1.27, 0.87–1.85) (Supplementary Table 12).

### Sensitivity analyses

Excluding dementia cases diagnosed within the first 5 years of follow-up did not materially alter the results (adjusted HR 1.46, 95% CI 1.34–1.59) (Supplementary Table 13). Risk of all-cause dementia remained significantly associated with baseline antidepressant use after further adjustment for baseline cognitive function (adjusted HR 1.42, 95% CI 1.21–1.68) (Supplementary Table 14). Similar results were observed when applying competing risk regression models (adjusted HR 1.45, 95% CI 1.33–1.57) (Supplementary Table 15) and in analyses using multiple imputation for missing data (adjusted HR 1.47, 95% CI 1.37–1.59) (Supplementary Table 16).

In addition, PSM was applied to create a matched cohort of baseline antidepressant users and non-users based on similar propensity scores. The matching process resulted in balanced baseline characteristics between the two groups, as indicated by SMDs of less than 0.1 for most covariates. However, some imbalance remained for depression (SMD = 0.151) and PHQ-2 total score (SMD = 0.164) (Supplementary Table 17). The adjusted HR for all-cause dementia in the matched cohort was 1.41 (95% CI 1.28–1.55) (Supplementary Table 18). IPTW was also applied to adjust for confounding by treatment assignment. The weighted adjusted HR for all-cause dementia in the IPTW analysis was 1.63 (95% CI 1.47–1.80) (Supplementary Table 19).

## Discussion

In this large prospective cohort study of over 460,000 UK Biobank participants, baseline antidepressant use was associated with a significantly increased risk of incident dementia over a mean follow-up of 13.4 years. This association remained after adjustment for a wide range of sociodemographic, lifestyle, health indicator, and antidepressant indication factors, as well as co-medication of other anticholinergics. The elevated risk was consistently observed across major dementia subtypes, including AD and VD.

In subsamples with cognitive testing and brain imaging data, baseline antidepressant use was further associated with poorer performance in fluid intelligence and prospective memory, as well as structural brain alterations. In the primary neuroimaging analysis, associations with lower total brain, lower gray matter volumes, lower gray matter volume in the hippocampus, smaller volumes of the basal nucleus, and greater white matter hyperintensity burden remained significant after Bonferroni correction. An inverse association with lateral orbitofrontal cortex volume was also observed, but did not remain significant after correction. These findings suggest that baseline antidepressant use is associated with both clinical and subclinical indicators of cognitive decline.

Our findings are consistent with several meta-analytic and cohort-based studies reporting an increased risk of dementia associated with antidepressant use. A meta-analysis by Chan et al. involving 18 longitudinal studies estimated a pooled RR of 1.37 (95% CI 1.11–1.70) for antidepressant users among depression patients (Lin et al., 2020). Similarly, Wang et al. found a pooled RR of 1.21 (95% CI 1.12-1.29) in older adults with depression (Wang et al., 2023). These findings suggest that antidepressant use may be associated with adverse long-term cognitive outcomes. Our study builds on this evidence by incorporating cognitive and neuroimaging data, providing additional context regarding the potential biological relevance of this association.

In contrast, the Rotterdam Study reported a positive but statistically non-significant association between antidepressant use and incident dementia (HR 1.14, 95% CI 0.92–1.41), which may partly reflect limited statistical power given its modest sample size (vom Hofe et al., 2024). By comparison, a UK Biobank study by Yang et al. observed an inverse association, suggesting reduced dementia risk among individuals receiving pharmacological and psychological treatments for late-life depression (Yang et al., 2023). Differences in exposure definition and cohort restriction may explain these discrepancies. Yang et al. incorporated historical primary care prescriptions dating back to 2000, while dementia follow-up began in 2006, potentially introducing immortal time bias, and excluded individuals with treatment-resistant depression, whereas our study aligned exposure assessment and follow-up at baseline and included the full spectrum of antidepressant users. These methodological differences likely contributed to heterogeneous findings across studies, while our larger cohort allowed this approach to be evaluated with greater precision.

Antidepressants are frequently prescribed for conditions beyond depression, including anxiety, insomnia, and chronic pain, all of which are independently linked to dementia risk (Kuring et al., 2020; Shi et al., 2018; Whitlock et al., 2017). This overlap complicates the disentanglement of medication effects from those underlying conditions. However, in our study, the association between baseline antidepressant use and dementia risk remained consistent across individuals with and without these indications, suggesting that the observed excess risk is unlikely to be fully explained by indication bias.

These findings are consistent with previous meta-analyses reporting increased dementia risk among antidepressant users in those with depression (RR 1.37, 95% CI 1.11–1.70) (Chan et al., 2019). A large Taiwanese cohort study also found elevated risk among users both with and without anxiety (HR 1.86 and 1.89), and sleep disorders (HR 2.11 and 1.88) (Lin et al., 2020). Although a stronger association was observed in individuals without depression (HR 1.89), but not in those with depression (HR 0.44, 95% CI 0.16–1.18), the latter estimate had a wide 95% CI, likely reflecting limited statistical power (Lin et al., 2020). Compared to these studies, our analysis leveraged a larger sample and more granular control for psychiatric and pain-related indications, providing further support that the observed association is not merely attributable to underlying clinical conditions.

We explored whether the association between baseline antidepressant use and dementia varied across key demographic subgroups. Statistically significant interaction effects were observed for age, education level, and living arrangement, with stronger associations found among participants younger than 60 years (HR 1.49), those with higher education (HR 1.67), and those not living alone (HR 1.54). These findings may reflect differences in medication adherence, treatment-seeking behavior, or access to health services. Individuals who are more engaged with their health care may undergo more frequent assessments and have cognitive decline identified earlier, which could increase dementia ascertainment and inflate observed associations (i.e., detection bias). Alternatively, individuals with greater social or cognitive reserve, which is often correlated with higher education and non-solitary living, may exhibit different vulnerabilities to pharmacological effects or underlying neurodegenerative processes, although the mechanisms remain uncertain (Stern, 2012).

While the interaction terms were statistically significant, the biological or behavioral mechanisms underlying these differences remain unclear. We did not find compelling evidence for effect reversal, and these patterns should be interpreted with caution. Nevertheless, they may help generate hypotheses regarding the identification of potentially vulnerable subgroups in whom baseline antidepressant use is associated with greater dementia risk, warranting closer monitoring or tailored management strategies.

Our study integrated cognitive and neuroimaging data to support the biological plausibility of the observed increased dementia risk associated with baseline antidepressant use. Baseline antidepressant users demonstrated lower performance in fluid intelligence and prospective memory tasks. This finding aligns with a large Swedish cohort study, where antidepressant use was associated with faster cognitive decline over time (e.g., *β* for escitalopram = −0.76 points/year, 95% CI −1.09 to −0.44), compared to non-users (Mo et al., 2025). Moreover, the dose–response relationship observed in that study, along with an increased risk of severe dementia and mortality at higher SSRI doses, further supports the potential adverse impact of antidepressants on long-term cognitive trajectories.

In our study, baseline antidepressant use was also negatively associated with neuroimaging markers of brain atrophy. These structural reductions are consistent with early neurodegenerative changes that often precede clinical cognitive impairment. Specifically, baseline antidepressant use was associated with reductions in both total brain volume and gray matter volume, which are global markers of brain atrophy and have been associated with cognitive decline (Fjell & Walhovd, 2010). The observed decrease in gray matter, particularly in the hippocampus, which is critical for episodic memory (Rao et al., 2022), suggests potential impairments in memory processes. Baseline antidepressant use was also associated with regional reductions in the basal nucleus, which is also implicated in cognitive function (Herrero et al., 2002). In addition, we observed a positive association between baseline antidepressant use and total white matter hyperintensity volume, a well-established marker of cerebral small vessel disease and chronic cerebrovascular injury (S. Zhang et al., 2023). This finding is consistent with previous studies indicating that antidepressants are independently associated with white matter lesions, particularly in patients with symptomatic atherosclerotic disease (Grool et al., 2013). Collectively, these findings are consistent with neurobiological patterns associated with dementia risk, although causal mechanisms cannot be inferred from the present analyses.

Although depression is widely recognized as a modifiable risk factor for dementia, our findings challenge the assumption that pharmacologically treating depressive symptoms necessarily reduces this risk. Antidepressants primarily modulate monoaminergic neurotransmission to alleviate mood symptoms (Cipriani et al., 2018). However, the biological pathways through which depression may increase dementia risk, such as small vessel disease and loss of behavioral-cognitive reserve, are not directly addressed by these pharmacological mechanisms.

We also revealed a positive association between baseline antidepressant use and small vessel disease markers, white matter hyperintensity. This finding is consistent with vascular pathway involvement, although it remains unclear whether this reflects medication-related effects, underlying disease burden, or shared vascular vulnerability. In addition, antidepressants, especially SSRIs, have been linked to clinically significant side effects relevant to dementia risk. A systematic review reported that up to 92% of individuals treated with SSRIs develop apathy, a symptom that emerges independently of mood improvement and across all age groups (Masdrakis, Markianos, & Baldwin, 2023). Apathy, characterized by reduced motivation and engagement in cognitively enriching or socially interactive activities, can accelerate the depletion of behavioral reserve and contribute to functional decline (Steffens, Fahed, Manning, & Wang, 2022). Longitudinal evidence also supports apathy as a mediator linking depressive symptoms to cognitive deterioration. For instance, a large cohort study in Parkinson’s disease found that within-person increases in apathy accounted for the association between depressive symptom fluctuations and declines in executive and memory performance (Szymkowicz et al., 2022).

Another proposed explanation is the anticholinergic hypothesis, as epidemiological studies have reported associations between exposure to anticholinergic antidepressants and an increased risk of dementia (Coupland et al., 2019; Richardson et al., 2018). However, in our analyses, both anticholinergic and non-anticholinergic antidepressants were associated with elevated risk of dementia compared with non-use, and the HR for anticholinergic agents was not greater than that for non-anticholinergics. This pattern indicates that anticholinergic effects alone are insufficient to fully explain the observed association.

Moreover, a recent systematic review and meta-analysis of randomized controlled trials (RCTs) examining antipsychotic (AP) drugs has provided further insight into the biological mechanisms at play. AP treatment has been shown to significantly disrupt glucose homeostasis, resulting in elevated levels of fasting glucose, insulin, HbA1c, and hyperglycemia risk, irrespective of weight gain (Smith et al., 2025). This disruption of glucose regulation is particularly concerning as diabetes is a well-established risk factor for dementia, especially through its effects on vascular health and inflammation (Livingston et al., 2024). The observed changes in glucose metabolism linked to AP use may, therefore, exacerbate the vascular pathophysiology contributing to dementia.

In addition, a recent study by Frank et al. demonstrated that certain midlife cognitive-affective depressive symptoms were associated with an increased risk of dementia, suggesting these symptoms could be early markers of underlying neurodegenerative processes (Frank et al., 2026). These findings support the idea that the prodrome of dementia might begin as early as midlife, long before the clinical diagnosis of dementia occurs. Given that depressive symptoms may reflect early neurodegeneration rather than merely a mood disorder, it is plausible that treating depression with antidepressants may not necessarily reduce the long-term risk of dementia. Instead, antidepressant therapy might alleviate symptoms without addressing the underlying neurodegenerative changes. This reinforces the hypothesis that depression in midlife may be a prodromal symptom of neurodegeneration, rather than a standalone risk factor for dementia.

Taken together, these findings indicate that while antidepressants may relieve affective symptoms, they are unlikely to modify the underlying neurodegenerative processes through which depression contributes to dementia. The findings suggest that pharmacological treatment is associated with a higher risk of dementia, highlighting the importance of judicious prescribing, regular cognitive monitoring, and the incorporation of non-pharmacological approaches. Regular reassessment of treatment indications may be warranted, and clinicians should remain attentive to potential cognitive changes during antidepressant therapy (Coupland et al., 2019; Kok & Reynolds, 2017). Furthermore, regular cognitive monitoring during antidepressant therapy may enable the early detection of cognitive decline and facilitate timely adjustment of treatment strategies (Koenig, Bhalla, & Butters, 2014).

Non-pharmacological approaches may complement pharmacological management in older adults with depression. Observational evidence has raised concerns about the long-term cognitive implications of pharmacotherapy (Wang et al., 2023), while population-based studies have reported that improvement after psychological therapy is associated with a lower incidence of later dementia among older adults with depression and anxiety disorders (John et al., 2023; Stott et al., 2023). Consistently, neuroimaging evidence indicates that CBT may be linked to increased gray matter volume in limbic regions (e.g., anterior hippocampus and amygdala), suggesting possible effects on brain plasticity (Zwiky et al., 2025).

This study benefited from several key strengths of the UK Biobank, including its large sample size, long-term follow-up, and comprehensive multidimensional data encompassing sociodemographic characteristics, lifestyle factors, psychiatric and medical history, and neurocognitive outcomes. These features enabled detailed adjustment for potential confounders and facilitated subgroup analyses across diverse clinical profiles. In addition, the integration of cognitive and neuroimaging markers provided additional support for the biological plausibility of the observed associations and helped mitigate concerns about reverse causation.

Several limitations should also be acknowledged. First, antidepressant use was self-reported at baseline, without information on treatment duration, dosage, clinical indication, or longitudinal changes. This precluded assessment of cumulative exposure and time-varying patterns of use, and may have led to exposure misclassification. This is particularly relevant over the long follow-up periods, where changes in treatment patterns could affect dementia risk. Although self-reported medication use has shown acceptable validity in epidemiological studies, self-reported antidepressant use showed good agreement between self-reported regular usage and prescription data (*k*: 0.85, 95% CI 0.84–0.87) (Hafferty et al., 2018). Nevertheless, repeated assessments over time would better capture longitudinal patterns and reduce exposure misclassification. Prior studies have evaluated potential dose–response relationships between antidepressant use and dementia risk based on cumulative exposure (days of exposure), underscoring the need for incorporating time-updated exposure data in future investigations (Ramos-Cejudo et al., 2024). In addition, our analysis was based on prevalent users of antidepressants at baseline rather than a new-user design. A new-user design would allow clearer temporal separation between treatment initiation and outcome occurrence, thereby further reducing potential reverse causation. However, this approach was not feasible in the present study because UK Biobank collected medication data at a single baseline assessment, precluding identification of incident antidepressant users. Future studies with repeated exposure measurements are warranted to address this limitation.

Second, although we adjusted for psychiatric diagnoses, symptom severity, and common indications for antidepressant use, including depression, anxiety, insomnia, and chronic pain, residual confounding by indication may remain. This is particularly relevant in cases where antidepressants were prescribed for prodromal cognitive symptoms or subtle behavioral changes not fully captured by available covariates. To address this, we performed sensitivity analyses using PSM and IPTW. All three approaches yielded consistent associations, but with different magnitudes: the covariate-adjusted model (HR 1.47) estimated the average treatment effect (ATE), PSM (HR 1.41) reflected the average treatment effect on the treated (ATT) in a more comparable subgroup, and IPTW (HR 1.63) amplified the influence of treated individuals with low propensity scores and higher dementia risk. Together, these findings support the robustness of our results while underscoring the distinct causal targets and limitations of each method. Additionally, while we used PHQ-2 as a proxy for mood disorder severity, it is a simplified tool and does not fully capture the severity or lifetime burden of mood disorders, such as would have been possible with PHQ-9 or other comprehensive measures. This limitation may contribute to unmeasured residual confounding, particularly in the assessment of long-term mood disorder burden.

Third, dementia diagnoses were ascertained via linkage to EHRs, which may lead to under-ascertainment of milder cases and potential misclassification of dementia subtypes. However, the classification algorithm employed has been validated within the UK Biobank and demonstrates acceptable accuracy (Wilkinson et al., 2019).

Fourth, despite excluding dementia cases occurring within the first 5 years of follow-up and incorporating cognitive and imaging data to strengthen temporal inference, reverse causation cannot be fully ruled out. While individuals with a dementia diagnosis at baseline were excluded, participants with early cognitive impairment could not be identified and removed. Such subclinical neurodegenerative processes may have simultaneously increased the likelihood of antidepressant use and accelerated subsequent cognitive decline. Nevertheless, the associations remained robust after further adjustment for baseline cognitive test scores, suggesting that reverse causation is unlikely to fully explain our findings. Furthermore, we analyzed cognitive performance and neuroimaging measures primarily as cross-sectional outcomes rather than modelling longitudinal rates of change, given the limited availability of repeated assessments. Future studies examining trajectories of cognitive decline and brain atrophy may provide additional insight into the temporal relationship between antidepressant use and neurodegeneration.

Fifth, residual confounding due to unmeasured or imperfectly captured factors such as frailty, healthcare-seeking behavior, and cognitive reserve may still have contributed to the observed associations. Although extensive covariate adjustment was performed, some level of uncontrolled confounding remains possible. In addition, differential healthcare contact among antidepressant users may increase the likelihood of dementia EHRs, introducing detection bias. Although direct measures of healthcare utilization were not available for adjustment, the consistency of findings across EHR-based dementia outcomes and independently collected cognitive and neuroimaging markers suggests that differential ascertainment alone is unlikely to fully explain the observed associations.

Finally, the UK Biobank cohort is not fully representative of the general population, as participants tend to be healthier and more educated. The healthy volunteer bias may be particularly relevant in our context, as individuals with severe depression may be less likely to participate or remain engaged in the study, potentially underrepresenting those at highest risk. In addition, survival bias may also be present. Given that our outcomes of interest, dementia, primarily occur in older adults, participants had to survive and remain healthy enough to attend the baseline assessment. These factors may limit the generalizability of absolute risk estimates, though internal validity and relative risk estimates are unlikely to be substantially biased.

In this large-scale, long-term cohort study, we found that baseline antidepressant use was associated with an increased risk of incident dementia, as well as with poorer cognitive performance and adverse structural brain changes. These findings underscore the importance of judicious prescribing, regular cognitive monitoring, and the incorporation of non-pharmacological approaches.

## Supporting information

10.1017/S0033291726104942.sm001 Liu et al. supplementary material Liu et al. supplementary material

## Data Availability

The current study was conducted using the UK Biobank resource under application No. 80476. All raw and derived data in this study are available from the UK Biobank (http://www.ukbiobank.ac.uk/).

## Supplementary material

The supplementary material for this article can be found at http://doi.org/10.1017/S0033291726104942.

## Long descriptions

### Figure 1. Long description

The flowchart begins at the top center with Total participants in UK Biobank N = 502,370.

An arrow points down to Eligible participants without history of dementia N = 502,157. To the right, a box indicates the exclusion of 213 participants with a history of dementia. To the left, a box links to Sensitivity analysis 4 regarding antidepressant use and dementia risk with multiple imputations N = 502,157.

A central arrow points down to Participants left in complete data analysis N = 461,464. To the right, a box details the exclusion of 40,693 participants missing covariates including Age, ethnicity, socioeconomic status, sleep duration, overall health rating, and PHQ-2 total score.

Below this, a vertical line connects several analysis categories on the left to their respective exclusion criteria on the right.

Primary analysis Table 2 and Subgroup analysis Supplementary Table 6 and 7 both involve N = 461,464.

Secondary analysis 1 Table 3 for cognition outcomes N = 57,330 excludes 404,134 participants without complete cognition testing data.

Secondary analysis 2 Figure 2 for neuroimaging outcomes N = 42,276 excludes 419,188 participants without complete neuroimaging data.

Exploratory analysis Supplementary Table 11 and 12 N = 460,001 excludes 1,463 participants using more than one antidepressant.

Sensitivity analysis 1 Supplementary Table 13 N = 460,829 excludes 635 participants diagnosed with dementia within the first 5 years.

Sensitivity analysis 2 Supplementary Table 14 N = 153,414 excludes 308,050 participants without complete baseline cognition data.

Sensitivity analysis 3 Supplementary Table 15 N = 461,464.

Sensitivity analysis 5 Supplementary Table 18 N = 67,442 excludes 394,022 participants unmatched based on propensity score matching.

Sensitivity analysis 6 Supplementary Table 19 N = 461,464.

Navigate back to Figure 1..

### Table 1. Long description

The table consists of five columns: Variable, All participants, Non-antidepressant use, Antidepressant use, and SMD.

Key demographic data includes:

- Total N: 461,464 total, with 427,743 (92.7 %) non-users and 33,721 (7.3 %) antidepressant users.

- Age: Mean of 57 years for all groups.

- Male: 47.0 percent in non-users versus 31.1 % in antidepressant users (SMD 0.331).

- Socioeconomic status: Antidepressant users show a higher percentage in the High category (25.3 %) compared to non-users (18.6 %).

Lifestyle and Health factors:

- Current smoking: 9.8 % in non-users versus 16.2 % in antidepressant users (SMD 0.189).

- Overall health: 18.0 % of non-users report excellent health compared to only 5.4 % of antidepressant users (SMD 0.735).

- Depression: 4.2 % in non-users versus 57.8 % in antidepressant users (SMD 1.422).

- PHQ-2 total score: 0.50 for non-users versus 1.54 for antidepressant users (SMD 0.740).

- Chronic pain: 41.1 % in non-users versus 65.4 % in antidepressant users (SMD 0.502).

- Other variables listed include White ethnicity, education level, living alone, alcohol consumption, physical inactivity, sleep duration, social isolation, hyperlipidemia, diabetes, hypertension, traumatic brain injury, hearing loss, vision loss, obesity, anxiety, insomnia, and use of other anticholinergics.

Note: SMD values greater than 0.1 are bolded in the original data to indicate significant differences.

Navigate back to Table 1..

### Table 2. Long description

The table compares a Non-antidepressant use group (N = 427,743) to an Antidepressant use group (N = 33,721).

For All-cause dementia:

* Number of events: 6,904 (1.6 %) for non-users vs 1,018 (3.0 %) for users.

* HR (95% CI) for Antidepressant use ranges from 2.13 [1.99, 2.28] in Model 1 to 1.47 [1.36, 1.60] in Model 5. All p-values are less than 0.001.

For Alzheimer’s disease:

* Number of events: 3,320 (0.8 %) for non-users vs 457 (1.4 %) for users.

* HR (95% CI) for Antidepressant use ranges from 1.93 [1.75, 2.13] in Model 1 to 1.53 [1.36, 1.73] in Model 5. All p-values are less than 0.001.

For Vascular dementia:

* Number of events: 1,584 (0.4 %) for non-users vs 275 (0.8 %) for users.

* HR (95% CI) for Antidepressant use ranges from 2.57 [2.25, 2.92] in Model 1 to 1.44 [1.23, 1.70] in Model 5. All p-values are less than 0.001.

Model adjustments:

* Model 1: Demographics.

* Model 2: Model 1 plus lifestyle factors.

* Model 3: Model 2 plus health indicators.

* Model 4: Model 3 plus antidepressant indication factors .

* Model 5: Model 4 plus other anticholinergics.

Navigate back to Table 2..

### Table 3. Long description

The table compares 54,433 non-antidepressant users to 2,897 antidepressant users.

Fluid intelligence score section:

* Mean (SD): 6.56 (2.06) for non-users and 6.27 (2.03) for users.

* β (95% CI) and p-values for antidepressant users relative to non-users (Ref):

* Model 1: -0.20 [-0.28, -0.13], p < 0.001.

* Model 2: -0.19 [-0.26, -0.12], p < 0.001.

* Model 3: -0.11 [-0.18, -0.03], p = 0.004.

* Model 4: -0.16 [-0.25, -0.08], p < 0.001.

* Model 5: -0.17 [-0.25, -0.08], p < 0.001.

Prospective memory result section:

* Positive prospective memory: 44,998 (82.7 %) for non-users and 2,311 (79.8 %) for users.

* OR (95% CI) and p-values for antidepressant users relative to non-users (Ref):

* Model 1: 0.80 [0.73, 0.88], p < 0.001.

* Model 2: 0.80 [0.73, 0.88], p < 0.001.

* Model 3: 0.84 [0.76, 0.92], p < 0.001.

* Model 4: 0.81 [0.72, 0.90], p < 0.001.

* Model 5: 0.81 [0.73, 0.91], p < 0.001.

Navigate back to Table 3..

### Figure 2. Long description

The forest plot consists of three columns: Neuroimaging outcomes, Differences (95% CI), and a graphical plot with a vertical dashed line at zero. For each outcome, Non-antidepressant use is the reference Ref.

* Volume of brain, grey plus white matter: Antidepressant use shows a difference (95% CI) of -0.058 [-0.099, -0.018]. Marked with a red asterisk.

* Volume of white matter: Antidepressant use shows a difference (95% CI) of -0.008 [-0.055, 0.039] .

* Volume of grey matter: Antidepressant use shows a difference (95% CI) of -0.082 [-0.119, -0.045] . Marked with a red asterisk.

* Total volume of white matter hyperintensities: Antidepressant use shows a difference (95% CI) of 0.131 [0.085, 0.177] . Marked with a red asterisk.

* Volume of hippocampus: Antidepressant use shows a difference (95% CI) of -0.044 [-0.089, 0.002] .

* Volume of grey matter in Hippocampus: Antidepressant use shows a difference (95% CI) of -0.079 [-0.124, -0.033] . Marked with a red asterisk.

* Volume of Cerebellum-Cortex: Antidepressant use shows a difference (95% CI) of -0.033 [-0.082, 0.015] .

* Volume of Basal-nucleus: Antidepressant use shows a difference (95% CI) of -0.115 [-0.162, -0.069] . Marked with a red asterisk.

* Volume of lateral orbitofrontal: Antidepressant use shows a difference (95% CI) of -0.050 [-0.097, -0.003] .

The x-axis of the plot ranges from negative 0.2 to 0.2. Data points to the left of the dashed line indicate lower volumes, while points to the right indicate higher volumes compared to the reference group.

Navigate back to Figure 2..
