# Supplementary material for: Antidepressant use and dementia, cognitive measures, and neuroimaging outcomes: A population-based cohort study
Source: Psychol Med. 2026 Jul 13;56:e225. doi: 10.1017/S0033291726104942 (PMC13370188; doi:10.1017/S0033291726104942)
Supplement: Liu et al. supplementary material [file S0033291726104942sup001.docx]

**Antidepressant use and dementia, cognitive measures, and neuroimaging outcomes in UK Biobank**

[Supplementary Table 1. Description of variables used in the present study. 3](#_Toc215750295)

[Supplementary Table 2. Criteria for defining self-reported medication use in Field 20003. 11](#_Toc215750296)

[Supplementary Table 3. Anticholinergic criteria for defining self-reported baseline antidepressant use in Field 20003. 17](#_Toc215750297)

[Supplementary Table 4. Baseline characteristics of all eligible participants and those with completed baseline data included in the main analysis. 23](#_Toc215750298)

[Supplementary Table 5. Number of incident dementia cases included in this study. 25](#_Toc215750299)

[Supplementary Table 6. Subgroup analysis for the association between antidepressant use and dementia across clinical indication subgroups 26](#_Toc215750300)

[Supplementary Table 7. Subgroup analysis for the association between antidepressant use and dementia across baseline demographic factors. 27](#_Toc215750301)

[Supplementary Table 8. Association of antidepressant use with normalized neuroimaging outcomes (z-score). 28](#_Toc215750302)

[Supplementary Table 9. Association of antidepressant use with normalized neuroimaging outcomes. 30](#_Toc215750303)

[Supplementary Table 10. Association of antidepressant use with crude neuroimaging outcomes. 32](#_Toc215750304)

[Supplementary Table 11. Risk of dementia associated with antidepressants in different anticholinergic activity. 34](#_Toc215750305)

[Supplementary Table 12. Risk of dementia associated with antidepressants in different pharmacological subtypes. 36](#_Toc215750306)

[Supplementary Table 13. Sensitivity analysis for association of antidepressant use with risk of dementia when excluding dementia case occurred in the first 5 years of follow-up. 37](#_Toc215750307)

[Supplementary Table 14. Sensitivity analysis for association of antidepressant use with risk of dementia among participants with cognitive function test in the baseline. 38](#_Toc215750308)

[Supplementary Table 15. Sensitivity analysis for association of antidepressant use with risk of dementia when using competing risk regression model. 39](#_Toc215750309)

[Supplementary Table 16. Sensitivity analysis for association of antidepressant use with risk of dementia with multiple imputations of missing data of covariates. 40](#_Toc215750310)

[Supplementary Table 17. Baseline characteristics of propensity score matching (PSM) matched participants. 41](#_Toc215750311)

[Supplementary Table 18. Sensitivity analysis for association of antidepressant use with risk of dementia based on propensity score matching (PSM). 42](#_Toc215750312)

[Supplementary Table 19. Sensitivity analysis for association of antidepressant use with risk of dementia based on inverse probability of treatment weighting (IPTW). 43](#_Toc215750313)

# Supplementary Table 1. Description of variables used in the present study.

| **Variables** | **Description** | **Reference group** |
| --- | --- | --- |
| **Sociodemographic variables** | | |
| Age | Continuous variable (Field 21022) |  |
| Sex | Categorical variable (male, female) (Field 31) | Female |
| Ethnicity | Categorical variable (white, non-white) (Field 21000) | White |
| Less education | Categorical variable (yes, no) (Field 6138, 10722, 845)  Education qualifications were collected though touchscreen questionnaire. If they did not have a college or university degree, they were asked what age they left full-time education. Participants without a degree and left full-time education before 21 years old or did not have data on the age at which they left education were coded as less education | No |
| Socioeconomic status | Categorical variable (low, intermediate, high) (Field 22189)  Townsend deprivation score, combining information on social class, employment, car availability and housing: categorized as quintiles 1 (low), 2 to 4 (intermediate) and 5 (high). | Low |
| Living alone status | Categorical variable (living alone, not living alone) (Field 709) | Not living alone |
| **Lifestyle behavior variables** | | |
| Excessive alcohol consumption | Categorical variable (without excessive alcohol consumption, with excessive alcohol consumption) (Field 4407, 4418, 4429, 4440, 4451, 4462, 1568, 1578, 1588, 1598, 1608, 5364, 1558, 20117)  Participants self-reported the number of alcohol units (10 ml of pure ethanol) consumed, in “units per week”, with above 21 units pre week were considered excessive alcohol consumption. | Without excessive alcohol consumption |
| Current smoking status | Categorical variable (Current smoking, non-current smoking)(Field 20116)  Current smokers were identified as current smoking. | Non-current smoking |
| Physical inactivity | Categorical variable (without physical inactivity, with physical inactivity) (Field 22032)  Participants in the “low” International Physical Activity Questionnaire (IPAQ) group were identified as with physical inactivity. | Without physical inactivity |
| Sleeping duration | Categorical variable (<7 hours, 7-8 hours, and ≥8 hours) (Field 1160) | < 7 hours |
| Social isolation | Categorical variable (without social isolation, with social isolation) (Field 709, 1031, 6160, 2020, 2110) Social isolation was defined using a composite measure derived from both objective and subjective indicators. Participants were classified as socially isolated if they either: (1) scored ≥2 on a 3-point social isolation scale; or (2) scored 2 on a 2-point loneliness scale assessing perceived loneliness and lack of confiding relationships. (Song 2023) | Without social isolation |
| **Health conditions variables** | | |
| Overall health | Categorical variable (Poor, fair, good, excellent) (Field 2178)  Self-rated overall health. | Poor |
| Hyperlipidemia | Categorical variable (No, yes) (Field 30780, 6177, 130814)  Identified by presence of direct-LDL>3 mmol/L, self-reported use of cholesterol lowing medication, or the earliest date of a diagnosis of ICD-10 disorders of lipoprotein metabolism and other lipidaemias (E78) in first occurrences record that prior the of baseline survey. | No |
| Diabetes | Categorical variable (No, yes) (Field 30750, 2443, 2976, 6177, 6153,130706, 130708, 130710, 130712, 130714)  Identified by presence of HbA1c >=6.5% (48 mmol/mol), self-reported diagnosed diabetes by a physician or age diabetes diagnosed, use of insulin and hypoglycemic medication, or the earliest date of a diagnosis of ICD-10 diabetes (E10-14) in first occurrences record that prior the of baseline survey. | No |
| Hypertension | Categorical variable (No, yes) (Field 4080, 93, 4079, 94, 6150, 2966, 6177, 6153, 131286, 131294)  Identified by SBP ≥140 mm Hg, DBP ≥90mmHg, self-reported diagnosed high blood pressure by a physician or age high blood pressure diagnosed, use of blood pressure medications, or the earliest date of a diagnosis of ICD-10 hypertension (I10, I15) in first occurrences record that prior the of baseline survey. | No |
| Traumatic brain injury | Categorical variable (No, yes) (Field 41271, 41281, 41270, 41280, 20002)  Defined by summary diagnosis of the hospital inpatient data using ICD-9 code (800-804, 850-854) and ICD-10 code (S00-S09), as well as self-reported illness code (1266, 1626) collected from a verbal interview. | No |
| Hearing loss | Categorical variable (No, yes) (Field 2247, 10793, 2257, 131258, 131260, 132460, 41270, 41271, 40001, 40002)  Identified by self-reported hearing difficulty, problems with background noise, self-report from ACE ("H90, H91, Q16"), and ICD diagnosis (death registration, hospital admission, and primary care). | No |
| Vision loss | Categorical variable (No, yes) (Field 5208, 5201, 5890, 6119, 5419, 5441, 5912, 5901, 4689, 5430, 4700, 5923, 5945, 5181, 5324, 5325, 5326, 5327, 5328, 131184, 131186, 131188, 131164, 131166, 131170, 131220, 41270, 41271, 40001, 40002) Identified by presenting distance visual acuity (VA) in the better-seeing eye worse than 0.3 logMAR (Snellen <20/40), based on logMAR, final measurements for left and right eyes, or by the presence of major eye diseases (cataract, glaucoma, diabetic eye disease, age-related macular degeneration) ascertained via self-report at baseline (questionnaire/ACE codes) or ICD codes (death, hospital admission, primary care) before baseline, including consideration of age at diagnosis relative to baseline age. | No |
| Obesity | Categorical variable (No, yes) (Field 21001)  Height and body weight were measured during the initial assessment center visit. Body mass index (BMI) was calculated as weight in kilograms (kg) divided by height in meters squared (m^2^) and participants were categorized as obese with BMI≥30 kg/m^2^. | No |
| **Antidepressant indication factors** | | |
| Depression | Categorical variable (Yes, no) (Field 130894, 130896)  The earliest date of a diagnosis of ICD-10 depression (F32-33) in first occurrences record that prior the of baseline survey. | No |
| PHQ-2 total score | Continuous variable (Field 2050, 2060)  The PHQ-2 total score, ranging from 0-6, was calculated based on two self-reported items assessing the frequency over the past two weeks of (1) feeling down, depressed, or hopeless and (2) having little interest or pleasure in doing things. Each item was scored from 0 to 3, corresponding to "not at all", "several days", "more than half the days", and "nearly every day". |  |
| Anxiety | Categorical variable (Yes, no) (Field 130904, 130906, 130908, 130910)  The earliest date of a diagnosis of ICD-10 anxiety disorder (F40-43) in first occurrences record that prior the of baseline survey. | No |
| Chronic pain | Categorical variable (Yes, no) (Field 2956, 3404, 3414, 3571, 3741, 3773, 3799, 4067)  Chronic pain was defined as self-reported having pain for at least 3 months in one or more of the following sites: head, face, neck/shoulder, back, abdomen, hip and knee.  Chronic widespread pain was defined as self-reported pain all over the body for at least 3 months. | No |
| Insomnia | Categorical variable (Yes, no) (Field 1200)  Self-reported difficulty initiating sleep or frequent nocturnal awakenings | No |
| **Co-medication factors** | | |
| Using other Anticholinergics | Categorical variable (No, yes) (Field 20003)  Self-reported regular medication taken weekly, monthly or three monthly were recorded in Field 20003, which were coded using 6,745 categories. According to a published study (Wu 2019), 1,809 of 6,745 categories were for medications taken by at least 10 participants, and 1,752 categories were successfully classified by active ingredients using the Anatomical Therapeutic Chemical (ATC) Classification System. We then identified the medication of interest among this 1752 categories by ATC information.  Anticholinergics besides antidepressants were identified according to a published review (Lavrador 2023), a list 130 types of drug with documented anticholinergic activity classified by ATC code was used among which 101 types were not antidepressants (ATC code ‘N06A antidepressants’). | No |
| **Antidepressant exposure variable** | Categorical variable (Non-antidepressant use, antidepressant use) (Field 20003)  Antidepressant use was identified through Field 20003 with ATC category ‘N06A Antidepressants’ | Non-antidepressant use |
| **Dementia outcome variables** | | |
| Dementia | Categorical variable (Yes, no) (Field 130836, 130838, 130840, 130842, 131036)  The earliest date of a diagnosis of ICD-10 dementia (F00-03, G30) in first occurrences record. | Yes |
| Alzheimer’s disease | Categorical variable (Yes, no) (Field 130836,131036)  The earliest date of a diagnosis of ICD-10 Alzheimer’s disease (F00, G30) in first occurrences record. | Yes |
| Vascular dementia | Categorical variable (Yes, no) (Field 130838)  The earliest date of a diagnosis of ICD-10 vascular dementia (F01) in first occurrences record. | Yes |
| **Cognition outcome variables** |  |  |
| Fluid intelligence score | Continuous variable (Field 20016)  Fluid intelligence, a 13-item task of problem-solving requiring logic and reasoning ability. |  |
| Prospective memory | Categorical variable (Correct, incorrect) (Field 20018)  Prospective memory, a type of episodic memory, measures an individual's memory for future tasks and was assessed via touch screen with a single instruction to be recalled later in the session. Values were coded as 0 for correct on the first attempt, and 1 otherwise. | Correct |
| **Neuroimaging outcomes** | Total brain volume (Field 25010), grey matter volume (Field 25006), white matter volume (Field 25008), hippocampal volume (sum of Field 25019 and Field 25020), lateral orbitofrontal volume (sum of Field 26900 and Field 26799), basal nucleus volume (sum of Field 26601and Field 26611), and white matter hyperintensities (Field 25781), which were ascertained using T1-weighted and T2-weighted fluid-attenuated inversion recovery volumes structural brain magnetic resonance imaging. All neuroimaging outcomes were scaled by head size (Field: 25000). |  |

**References**:

Song Y, Zhu C, Shi B, Song C, Cui K, Chang Z, et al. Social isolation, loneliness, and incident type 2 diabetes mellitus: results from two large prospective cohorts in Europe and East Asia and Mendelian randomization. EClinicalMedicine. 2023;64:102236.

Wu Y, Byrne EM, Zheng Z, Kemper KE, Yengo L, Mallett AJ, et al. Genome-wide association study of medication-use and associated disease in the UK Biobank. Nature Communications. 2019;10(1):1891.

Lavrador M, Cabral AC, Veríssimo MT, Fernandez-Llimos F, Figueiredo IV, Castel-Branco MM. A Universal Pharmacological-Based List of Drugs with Anticholinergic Activity. Pharmaceutics. 2023;15(1).

# Supplementary Table 2. Criteria for defining self-reported medication use in Field 20003.

| **No** | **Category** | | **Coding** | **Medication ATC code** | **Drug name** | **Notes*** |
| --- | --- | --- | --- | --- | --- | --- |
| 1 | amitriptyline | | 1140879616 | N06AA09 | Amitriptyline | Antidepressants |
| 2 | citalopram | | 1140921600 | N06AB04 | Citalopram | Antidepressants |
| 3 | fluoxetine | | 1140879540 | N06AB03 | Fluoxetine | Antidepressants |
| 4 | sertraline | | 1140867878 | N06AB06 | Sertraline | Antidepressants |
| 5 | venlafaxine | | 1140916282 | N06AX16 | Venlafaxine | Antidepressants |
| 6 | dosulepin | | 1140909806 | N06AA16 | Dosulepin | Antidepressants |
| 7 | paroxetine | | 1140867888 | N06AB05 | Paroxetine | Antidepressants |
| 8 | mirtazapine | | 1141152732 | N06AX11 | Mirtazapine | Antidepressants |
| 9 | escitalopram | | 1141180212 | N06AB10 | Escitalopram | Antidepressants |
| 10 | st john's wort/hypericum [ctsu] | | 1201 | N06AX25 | St. John'S Wort | Antidepressants |
| 11 | trazodone | | 1140879634 | N06AX05 | Trazodone | Antidepressants |
| 12 | prozac 20mg capsule | | 1140867876 | N06AB03 | Fluoxetine | Antidepressants |
| 13 | seroxat 20mg tablet | | 1140882236 | N06AB05 | Paroxetine | Antidepressants |
| 14 | duloxetine | | 1141200564 | N06AX21 | Duloxetine | Antidepressants |
| 15 | cipralex 5mg tablet | | 1141190158 | N06AB10 | Escitalopram | Antidepressants |
| 16 | lofepramine | | 1140867726 | N06AA07 | Lofepramine | Antidepressants |
| 17 | clomipramine | | 1140879620 | N06AA04 | Clomipramine | Antidepressants |
| 18 | nortriptyline | | 1140867818 | N06AA10 | Nortriptyline | Antidepressants |
| 19 | imipramine | | 1140879630 | N06AA02 | Imipramine | Antidepressants |
| 20 | efexor 37.5mg tablet | | 1140916288 | N06AX16 | Venlafaxine | Antidepressants |
| 21 | dothiepin | | 1140879628 | N06AA16 | Dosulepin | Antidepressants |
| 22 | cipramil 10mg tablet | | 1141151946 | N06AB04 | Citalopram | Antidepressants |
| 23 | prothiaden 25mg capsule | | 1140867624 | N06AA16 | Dosulepin | Antidepressants |
| 24 | trimipramine | | 1140867756 | N06AA06 | Trimipramine | Antidepressants |
| 25 | lustral 50mg tablet | | 1140867884 | N06AB06 | Sertraline | Antidepressants |
| 26 | reboxetine | | 1141151978 | N06AX18 | Reboxetine | Antidepressants |
| 27 | zispin 30mg tablet | | 1141152736 | N06AX11 | Mirtazapine | Antidepressants |
| 28 | cymbalta 30mg gastro-resistant capsule | | 1141201834 | N06AX21 | Duloxetine | Antidepressants |
| 29 | anafranil 10mg capsule | | 1140867690 | N06AA04 | Clomipramine | Antidepressants |
| 30 | doxepin | | 1140867640 | N06AA12 | Doxepin | Antidepressants |
| 31 | moclobemide | | 1140867920 | N06AG02 | Moclobemide | Antidepressants |
| 32 | yentreve 20mg gastro-resistant capsule | | 1141200570 | N06AX21 | Duloxetine | Antidepressants |
| 33 | phenelzine | | 1140867850 | N06AF03 | Phenelzine | Antidepressants |
| 34 | fluvoxamine | | 1140879544 | N06AB08 | Fluvoxamine | Antidepressants |
| 35 | oxactin 20mg capsule | | 1141174756 | N06AB03 | Fluoxetine | Antidepressants |
| 36 | surmontil 10mg tablet | | 1140867758 | N06AA06 | Trimipramine | Antidepressants |
| 37 | tryptophan product | | 1140867960 | N06AX02 | Tryptophan | Antidepressants |
| 38 | zyban 150mg m/r tablet | | 1141176858 | N06AX12 | Bupropion | Antidepressants |
| 39 | tranylcypromine | | 1140867914 | N06AF04 | Tranylcypromine | Antidepressants |
| 40 | bupropion | | 1141176854 | N06AX12 | Bupropion | Antidepressants |
| 41 | allegron 10mg tablet | | 1140867820 | N06AA10 | Nortriptyline | Antidepressants |
| 42 | tramadol | | 1140864992 | N02AX02 | Tramadol* | Other anticholinergics |
| 43 | loratadine | | 1140862772 | R06AX13 | Loratadine | Other anticholinergics |
| 44 | mebeverine | | 1140879428 | A03AA04 | Mebeverine | Other anticholinergics |
| 45 | tiotropium | | 1141182628 | R03BB04 | Tiotropium | Other anticholinergics |
| 46 | oxybutynin | | 1140883568 | G04BD04 | Oxybutynin | Other anticholinergics |
| 47 | tolterodine l-tartrate | | 1141162764 | G04BD07 | Tolterodine | Other anticholinergics |
| 48 | solifenacin | | 1141200322 | G04BD08 | Solifenacin | Other anticholinergics |
| 49 | detrusitol 1mg tablet | | 1141162824 | G04BD07 | Tolterodine | Other anticholinergics |
| 50 | | spiriva 18micrograms inhalation capsule | 1141182632 | R03BB04 | Tiotropium | Other anticholinergics |
| 51 | prochlorperazine | | 1140868170 | N05AB04 | Prochlorperazine | Other anticholinergics |
| 52 | colofac-100 tablet | | 1141167334 | A03AA04 | Mebeverine | Other anticholinergics |
| 53 | olanzapine | | 1140928916 | N05AH03 | Olanzapine | Other anticholinergics |
| 54 | ipratropium | | 1140883548 | R01AX03 \|R03BB01 | Ipratropium Bromide | Other anticholinergics |
| 55 | vesicare 5mg tablet | | 1141200384 | G04BD08 | Solifenacin | Other anticholinergics |
| 56 | atrovent 20micrograms inhaler | | 1140862236 | R03BB01 | Ipratropium Bromide | Other anticholinergics |
| 57 | buscopan 10mg tablet | | 1140865396 | A03BB01 | Scopolamine Butylbromide | Other anticholinergics |
| 58 | etoricoxib | | 1141180140 | M01AH05 | Etoricoxib** | Other anticholinergics |
| 59 | amiodarone | | 1140888502 | C01BD01 | Amiodarone | Other anticholinergics |
| 60 | clarityn 10mg tablet | | 1140862776 | R06AX13 | Loratadine | Other anticholinergics |
| 61 | quetiapine | | 1141152848 | N05AH04 | Quetiapine | Other anticholinergics |
| 62 | stemetil 5mg tablet | | 1140868172 | N05AB04 | Prochlorperazine | Other anticholinergics |
| 63 | risperidone | | 1140867444 | N05AX08 | Risperidone | Other anticholinergics |
| 64 | cinnarizine | | 1140883524 | N07CA02 | Cinnarizine | Other anticholinergics |
| 65 | trospium | | 1141171496 | G04BD09 | Trospium | Other anticholinergics |
| 66 | procyclidine | | 1140883476 | N04AA04 | Procyclidine | Other anticholinergics |
| 67 | arcoxia 60mg tablet | | 1141180148 | M01AH05 | Etoricoxib** | Other anticholinergics |
| 68 | chlorpromazine | | 1140879658 | N05AA01 | Chlorpromazine | Other anticholinergics |
| 69 | hydroxyzine | | 1140883656 | N05BB01 | Hydroxyzine | Other anticholinergics |
| 70 | hyoscine butylbromide | | 1140865394 | A03BB01 | Scopolamine Butylbromide | Other anticholinergics |
| 71 | merbentyl 10mg tablet | | 1140865382 | A03AA07 | Dicyclomine | Other anticholinergics |
| 72 | arcoxia 90mg tablet | | 1141180150 | M01AH05 | Etoricoxib** | Other anticholinergics |
| 73 | zydol 50mg capsule | | 1140865000 | N02AX02 | Tramadol* | Other anticholinergics |
| 74 | buccastem 3mg tablet | | 1140868192 | N05AB04 | Prochlorperazine | Other anticholinergics |
| 75 | fentanyl | | 1140880956 | N01AH01 \|N02AB03 | Fentanyl* | Other anticholinergics |
| 76 | loratadine product | | 1141157324 | R06AX13 | Loratadine | Other anticholinergics |
| 77 | trifluoperazine | | 1140868120 | N05AB06 | Trifluoperazine | Other anticholinergics |
| 78 | phenergan 10mg tablet | | 1140862810 | R06AD02 | Promethazine | Other anticholinergics |
| 79 | dicycloverine | | 1141194852 | A03AA07 | Dicyclomine | Other anticholinergics |
| 80 | promethazine product | | 1140882082 | D04AA10 \|R06AD02 | Promethazine | Other anticholinergics |
| 81 | nytol 25mg tablet | | 1140862722 | R06AA02 | Diphenhydramine | Other anticholinergics |
| 82 | fentanyl product | | 1141157470 | N01AH01 \|N02AB03 | Fentanyl* | Other anticholinergics |
| 83 | stugeron 15mg tablet | | 1140868074 | N07CA02 | Cinnarizine | Other anticholinergics |
| 84 | ditropan 2.5mg tablet | | 1140869492 | G04BD04 | Oxybutynin | Other anticholinergics |
| 85 | arcoxia 120mg tablet | | 1141180152 | M01AH05 | Etoricoxib** | Other anticholinergics |
| 86 | trihexyphenidyl | | 1140909816 | N04AA01 | Trihexyphenidyl | Other anticholinergics |
| 87 | lyrinel xl 5mg m/r tablet | | 1141193290 | G04BD04 | Oxybutynin | Other anticholinergics |
| 88 | seroquel 25mg tablet | | 1141152860 | N05AH04 | Quetiapine | Other anticholinergics |
| 89 | benadryl 25mg capsule | | 1140855562 | R06AA02 | Diphenhydramine | Other anticholinergics |
| 90 | aripiprazole | | 1141195974 | N05AX12 | Aripiprazole | Other anticholinergics |
| 91 | haloperidol | | 1140867168 | N05AD01 | Haloperidol | Other anticholinergics |
| 92 | atarax 10mg tablet | | 1140863286 | N05BB01 | Hydroxyzine | Other anticholinergics |
| 93 | stelazine 1mg tablet | | 1140867244 | N05AB06 | Trifluoperazine | Other anticholinergics |
| 94 | regurin 20mg tablet | | 1141171502 | G04BD09 | Trospium | Other anticholinergics |
| 95 | depixol 3mg tablet | | 1140867152 | N05AF01 | Flupentixol | Other anticholinergics |
| 96 | hyoscine | | 1140882104 | A04AD01 \|N05CM05 \|S01FA02 | Scopolamine | Other anticholinergics |
| 97 | flupentixol | | 1140909800 | N05AF01 | Flupentixol | Other anticholinergics |
| 98 | mebeverine hcl+ispaghula 135mg/3.5g/sachet granules | | 1140865408 | A06AC01 \|A03AA04 | Plantago Seed \|Mebeverine Hydrochloride | Other anticholinergics |
| 99 | propiverine hydrochloride | | 1141168714 | G04BD06 | Propiverine | Other anticholinergics |
| 100 | clozapine | | 1140867420 | N05AH02 | Clozapine | Other anticholinergics |
| 101 | nytol 25mg caplet | | 1141153080 | R06AA02 | Diphenhydramine | Other anticholinergics |
| 102 | detrunorm 15mg tablet | | 1141168718 | G04BD06 | Propiverine | Other anticholinergics |
| 103 | ucerax 25mg tablet | | 1140863292 | N05BB01 | Hydroxyzine | Other anticholinergics |
| 104 | propantheline | | 1140883480 | A03AB05 | Propantheline | Other anticholinergics |
| 105 | promazine | | 1140879746 | N05AA03 | Promazine | Other anticholinergics |
| 106 | risperdal 0.5mg tablet | | 1141177762 | N05AX08 | Risperidone | Other anticholinergics |
| 107 | diphenhydramine product | | 1140883600 | D04AA32 \|R06AA02 | Diphenhydramine | Other anticholinergics |
| 108 | zamadol 50mg capsule | | 1140928742 | N02AX02 | Tramadol* | Other anticholinergics |
| 109 | modecate 12.5mg/0.5ml oily injection | | 1140867456 | N05AB02 | Fluphenazine | Other anticholinergics |
| 110 | fluanxol 500micrograms tablet | | 1140867952 | N05AF01 | Flupentixol | Other anticholinergics |
| 111 | disopyramide | | 1140866568 | C01BA03 | Disopyramide | Other anticholinergics |
| 112 | durogesic 25micrograms/hour patch | | 1140911830 | N02AB03 | Fentanyl* | Other anticholinergics |
| 113 | quinidine | | 1140851530 | C01BA01 | Quinidine | Other anticholinergics |
| 114 | orphenadrine | | 1140883560 | N04AB02 \|M03BC01 | Orphenadrine | Other anticholinergics |
| 115 | vallergan 10mg tablet | | 1140862742 | R06AD01 | Alimemazine | Other anticholinergics |
| 116 | zamadol sr 100mg m/r capsule | | 1141153424 | N02AX02 | Tramadol* | Other anticholinergics |
| 117 | pro-banthine 15mg tablet | | 1140865330 | A03AB05 | Propantheline | Other anticholinergics |
| 118 | flupenthixol | | 1140867150 | N05AF01 | Flupentixol | Other anticholinergics |
| 119 | atropine | | 1140883494 | A03BA01 \|S01FA01 | Atropine | Other anticholinergics |
| 120 | alimemazine | | 1140909792 | R06AD01 | Alimemazine | Other anticholinergics |
| 121 | zydol sr 100mg m/r tablet | | 1140922636 | N02AX02 | Tramadol* | Other anticholinergics |
| 122 | pethidine | | 1140884388 | N02AB02 | Pethidine* | Other anticholinergics |
| 123 | zyprexa 2.5mg tablet | | 1141167976 | N05AH03 | Olanzapine | Other anticholinergics |
| 124 | flavoxate | | 1140883914 | G04BD02 | Flavoxate | Other anticholinergics |
| 125 | ketotifen | | 1140862668 | R06AX17 \|S01GX08 | Ketotifen | Other anticholinergics |
| 126 | durogesic 50micrograms/hour patch | | 1140911832 | N02AB03 | Fentanyl* | Other anticholinergics |
| 127 | durogesic 75micrograms/hour patch | | 1140911834 | N02AB03 | Fentanyl* | Other anticholinergics |
| 128 | largactil 10mg tablet | | 1140863416 | N05AA01 | Chlorpromazine | Other anticholinergics |
| 129 | ditropan xl 5mg m/r tablet | | 1141177080 | G04BD04 | Oxybutynin | Other anticholinergics |
| 130 | hyoscine product | | 1141157482 | A04AD01 \|N05CM05 \|S01FA02 | Scopolamine | Other anticholinergics |
| 131 | dicyclomine hydrochloride | | 1140865378 | A03AA07 | Dicyclomine | Other anticholinergics |
| 132 | abilify 5mg tablet | | 1141202024 | N05AX12 | Aripiprazole | Other anticholinergics |
| 133 | clemastine | | 1140862638 | D04AA14 \|R06AA04 | Clemastine | Other anticholinergics |
| 134 | serenace 500micrograms capsule | | 1140867092 | N05AD01 | Haloperidol | Other anticholinergics |
| 135 | haldol 5mg tablet | | 1140867184 | N05AD01 | Haloperidol | Other anticholinergics |
| 136 | fluphenazine | | 1140882098 | N05AB02 | Fluphenazine | Other anticholinergics |
| 137 | durogesic 100micrograms/hour patch | | 1140911836 | N02AB03 | Fentanyl* | Other anticholinergics |
| 138 | zydol soluble 50mg tablet | | 1140928372 | N02AX02 | Morphine* | Other anticholinergics |
| 139 | cyclopentolate | | 1140883518 | S01FA04 | Cyclopentolate | Other anticholinergics |
| 140 | piportil depot 50mg/1ml oily injection | | 1140867572 | N05AC04 | Pipotiazine | Other anticholinergics |
| 141 | steri-neb ipratropium 250micrograms/1ml nebulisation unit | | 1140888906 | R03BB01 | Ipratropium Bromide | Other anticholinergics |
| 142 | clozaril 25mg tablet | | 1140882320 | N05AH02 | Clozapine | Other anticholinergics |

# Supplementary Table 3. Anticholinergic criteria for defining self-reported baseline antidepressant use in Field 20003.

| **Anticholinergic criteria** | **No** | **Category** | **Coding** | **Medication ATC code** | **Drug name** | **Notes*** |
| --- | --- | --- | --- | --- | --- | --- |
| (Coupland et al., 2019) | 1 | amitriptyline | 1140879616 | N06AA09 | Amitriptyline | Anticholinergic |
|  | 2 | citalopram | 1140921600 | N06AB04 | Citalopram | Non-anticholinergic |
|  | 3 | fluoxetine | 1140879540 | N06AB03 | Fluoxetine | Non-anticholinergic |
|  | 4 | sertraline | 1140867878 | N06AB06 | Sertraline | Non-anticholinergic |
|  | 5 | venlafaxine | 1140916282 | N06AX16 | Venlafaxine | Non-anticholinergic |
|  | 6 | dosulepin | 1140909806 | N06AA16 | Dosulepin | Anticholinergic |
|  | 7 | paroxetine | 1140867888 | N06AB05 | Paroxetine | Anticholinergic |
|  | 8 | mirtazapine | 1141152732 | N06AX11 | Mirtazapine | Non-anticholinergic |
|  | 9 | escitalopram | 1141180212 | N06AB10 | Escitalopram | Non-anticholinergic |
|  | 10 | st john's wort/hypericum [ctsu] | 1201 | N06AX25 | St. John'S Wort | Non-anticholinergic |
|  | 11 | trazodone | 1140879634 | N06AX05 | Trazodone | Non-anticholinergic |
|  | 12 | prozac 20mg capsule | 1140867876 | N06AB03 | Fluoxetine | Non-anticholinergic |
|  | 13 | seroxat 20mg tablet | 1140882236 | N06AB05 | Paroxetine | Anticholinergic |
|  | 14 | duloxetine | 1141200564 | N06AX21 | Duloxetine | Non-anticholinergic |
|  | 15 | cipralex 5mg tablet | 1141190158 | N06AB10 | Escitalopram | Non-anticholinergic |
|  | 16 | lofepramine | 1140867726 | N06AA07 | Lofepramine | Anticholinergic |
|  | 17 | clomipramine | 1140879620 | N06AA04 | Clomipramine | Anticholinergic |
|  | 18 | nortriptyline | 1140867818 | N06AA10 | Nortriptyline | Anticholinergic |
|  | 19 | imipramine | 1140879630 | N06AA02 | Imipramine | Anticholinergic |
|  | 20 | efexor 37.5mg tablet | 1140916288 | N06AX16 | Venlafaxine | Non-anticholinergic |
|  | 21 | dothiepin | 1140879628 | N06AA16 | Dosulepin | Anticholinergic |
|  | 22 | cipramil 10mg tablet | 1141151946 | N06AB04 | Citalopram | Non-anticholinergic |
|  | 23 | prothiaden 25mg capsule | 1140867624 | N06AA16 | Dosulepin | Anticholinergic |
|  | 24 | trimipramine | 1140867756 | N06AA06 | Trimipramine | Anticholinergic |
|  | 25 | lustral 50mg tablet | 1140867884 | N06AB06 | Sertraline | Non-anticholinergic |
|  | 26 | reboxetine | 1141151978 | N06AX18 | Reboxetine | Non-anticholinergic |
|  | 27 | zispin 30mg tablet | 1141152736 | N06AX11 | Mirtazapine | Non-anticholinergic |
|  | 28 | cymbalta 30mg gastro-resistant capsule | 1141201834 | N06AX21 | Duloxetine | Non-anticholinergic |
|  | 29 | anafranil 10mg capsule | 1140867690 | N06AA04 | Clomipramine | Anticholinergic |
|  | 30 | doxepin | 1140867640 | N06AA12 | Doxepin | Anticholinergic |
|  | 31 | moclobemide | 1140867920 | N06AG02 | Moclobemide | Non-anticholinergic |
|  | 32 | yentreve 20mg gastro-resistant capsule | 1141200570 | N06AX21 | Duloxetine | Non-anticholinergic |
|  | 33 | phenelzine | 1140867850 | N06AF03 | Phenelzine | Non-anticholinergic |
|  | 34 | fluvoxamine | 1140879544 | N06AB08 | Fluvoxamine | Non-anticholinergic |
|  | 35 | oxactin 20mg capsule | 1141174756 | N06AB03 | Fluoxetine | Non-anticholinergic |
|  | 36 | surmontil 10mg tablet | 1140867758 | N06AA06 | Trimipramine | Anticholinergic |
|  | 37 | tryptophan product | 1140867960 | N06AX02 | Tryptophan | Non-anticholinergic |
|  | 38 | zyban 150mg m/r tablet | 1141176858 | N06AX12 | Bupropion | Non-anticholinergic |
|  | 39 | tranylcypromine | 1140867914 | N06AF04 | Tranylcypromine | Non-anticholinergic |
|  | 40 | bupropion | 1141176854 | N06AX12 | Bupropion | Non-anticholinergic |
|  | 41 | allegron 10mg tablet | 1140867820 | N06AA10 | Nortriptyline | Anticholinergic |
| (Richardson et al., 2018) | 1 | amitriptyline | 1140879616 | N06AA09 | Amitriptyline | Anticholinergic |
|  | 2 | citalopram | 1140921600 | N06AB04 | Citalopram | Anticholinergic |
|  | 3 | fluoxetine | 1140879540 | N06AB03 | Fluoxetine | Non-anticholinergic |
|  | 4 | sertraline | 1140867878 | N06AB06 | Sertraline | Non-anticholinergic |
|  | 5 | venlafaxine | 1140916282 | N06AX16 | Venlafaxine | Non-anticholinergic |
|  | 6 | dosulepin | 1140909806 | N06AA16 | Dosulepin | Anticholinergic |
|  | 7 | paroxetine | 1140867888 | N06AB05 | Paroxetine | Anticholinergic |
|  | 8 | mirtazapine | 1141152732 | N06AX11 | Mirtazapine | Non-anticholinergic |
|  | 9 | escitalopram | 1141180212 | N06AB10 | Escitalopram | Non-anticholinergic |
|  | 10 | st john's wort/hypericum [ctsu] | 1201 | N06AX25 | St. John'S Wort | Non-anticholinergic |
|  | 11 | trazodone | 1140879634 | N06AX05 | Trazodone | Non-anticholinergic |
|  | 12 | prozac 20mg capsule | 1140867876 | N06AB03 | Fluoxetine | Non-anticholinergic |
|  | 13 | seroxat 20mg tablet | 1140882236 | N06AB05 | Paroxetine | Anticholinergic |
|  | 14 | duloxetine | 1141200564 | N06AX21 | Duloxetine | Non-anticholinergic |
|  | 15 | cipralex 5mg tablet | 1141190158 | N06AB10 | Escitalopram | Non-anticholinergic |
|  | 16 | lofepramine | 1140867726 | N06AA07 | Lofepramine | Anticholinergic |
|  | 17 | clomipramine | 1140879620 | N06AA04 | Clomipramine | Anticholinergic |
|  | 18 | nortriptyline | 1140867818 | N06AA10 | Nortriptyline | Anticholinergic |
|  | 19 | imipramine | 1140879630 | N06AA02 | Imipramine | Anticholinergic |
|  | 20 | efexor 37.5mg tablet | 1140916288 | N06AX16 | Venlafaxine | Non-anticholinergic |
|  | 21 | dothiepin | 1140879628 | N06AA16 | Dosulepin | Anticholinergic |
|  | 22 | cipramil 10mg tablet | 1141151946 | N06AB04 | Citalopram | Anticholinergic |
|  | 23 | prothiaden 25mg capsule | 1140867624 | N06AA16 | Dosulepin | Anticholinergic |
|  | 24 | trimipramine | 1140867756 | N06AA06 | Trimipramine | Anticholinergic |
|  | 25 | lustral 50mg tablet | 1140867884 | N06AB06 | Sertraline | Non-anticholinergic |
|  | 26 | reboxetine | 1141151978 | N06AX18 | Reboxetine | Non-anticholinergic |
|  | 27 | zispin 30mg tablet | 1141152736 | N06AX11 | Mirtazapine | Non-anticholinergic |
|  | 28 | cymbalta 30mg gastro-resistant capsule | 1141201834 | N06AX21 | Duloxetine | Non-anticholinergic |
|  | 29 | anafranil 10mg capsule | 1140867690 | N06AA04 | Clomipramine | Anticholinergic |
|  | 30 | doxepin | 1140867640 | N06AA12 | Doxepin | Non-anticholinergic |
|  | 31 | moclobemide | 1140867920 | N06AG02 | Moclobemide | Non-anticholinergic |
|  | 32 | yentreve 20mg gastro-resistant capsule | 1141200570 | N06AX21 | Duloxetine | Non-anticholinergic |
|  | 33 | phenelzine | 1140867850 | N06AF03 | Phenelzine | Non-anticholinergic |
|  | 34 | fluvoxamine | 1140879544 | N06AB08 | Fluvoxamine | Non-anticholinergic |
|  | 35 | oxactin 20mg capsule | 1141174756 | N06AB03 | Fluoxetine | Non-anticholinergic |
|  | 36 | surmontil 10mg tablet | 1140867758 | N06AA06 | Trimipramine | Anticholinergic |
|  | 37 | tryptophan product | 1140867960 | N06AX02 | Tryptophan | Non-anticholinergic |
|  | 38 | zyban 150mg m/r tablet | 1141176858 | N06AX12 | Bupropion | Non-anticholinergic |
|  | 39 | tranylcypromine | 1140867914 | N06AF04 | Tranylcypromine | Non-anticholinergic |
|  | 40 | bupropion | 1141176854 | N06AX12 | Bupropion | Non-anticholinergic |
|  | 41 | allegron 10mg tablet | 1140867820 | N06AA10 | Nortriptyline | Anticholinergic |
| (Lavrador et al., 2023) | 1 | amitriptyline | 1140879616 | N06AA09 | Amitriptyline | Anticholinergic |
|  | 2 | citalopram | 1140921600 | N06AB04 | Citalopram | Anticholinergic |
|  | 3 | fluoxetine | 1140879540 | N06AB03 | Fluoxetine | Anticholinergic |
|  | 4 | sertraline | 1140867878 | N06AB06 | Sertraline | Anticholinergic |
|  | 5 | venlafaxine | 1140916282 | N06AX16 | Venlafaxine | Non-anticholinergic |
|  | 6 | dosulepin | 1140909806 | N06AA16 | Dosulepin | Anticholinergic |
|  | 7 | paroxetine | 1140867888 | N06AB05 | Paroxetine | Anticholinergic |
|  | 8 | mirtazapine | 1141152732 | N06AX11 | Mirtazapine | Anticholinergic |
|  | 9 | escitalopram | 1141180212 | N06AB10 | Escitalopram | Anticholinergic |
|  | 10 | st john's wort/hypericum [ctsu] | 1201 | N06AX25 | St. John'S Wort | Non-anticholinergic |
|  | 11 | trazodone | 1140879634 | N06AX05 | Trazodone | Non-anticholinergic |
|  | 12 | prozac 20mg capsule | 1140867876 | N06AB03 | Fluoxetine | Anticholinergic |
|  | 13 | seroxat 20mg tablet | 1140882236 | N06AB05 | Paroxetine | Anticholinergic |
|  | 14 | duloxetine | 1141200564 | N06AX21 | Duloxetine | Anticholinergic |
|  | 15 | cipralex 5mg tablet | 1141190158 | N06AB10 | Escitalopram | Anticholinergic |
|  | 16 | lofepramine | 1140867726 | N06AA07 | Lofepramine | Anticholinergic |
|  | 17 | clomipramine | 1140879620 | N06AA04 | Clomipramine | Anticholinergic |
|  | 18 | nortriptyline | 1140867818 | N06AA10 | Nortriptyline | Anticholinergic |
|  | 19 | imipramine | 1140879630 | N06AA02 | Imipramine | Anticholinergic |
|  | 20 | efexor 37.5mg tablet | 1140916288 | N06AX16 | Venlafaxine | Non-anticholinergic |
|  | 21 | dothiepin | 1140879628 | N06AA16 | Dosulepin | Anticholinergic |
|  | 22 | cipramil 10mg tablet | 1141151946 | N06AB04 | Citalopram | Anticholinergic |
|  | 23 | prothiaden 25mg capsule | 1140867624 | N06AA16 | Dosulepin | Anticholinergic |
|  | 24 | trimipramine | 1140867756 | N06AA06 | Trimipramine | Anticholinergic |
|  | 25 | lustral 50mg tablet | 1140867884 | N06AB06 | Sertraline | Anticholinergic |
|  | 26 | reboxetine | 1141151978 | N06AX18 | Reboxetine | Non-anticholinergic |
|  | 27 | zispin 30mg tablet | 1141152736 | N06AX11 | Mirtazapine | Anticholinergic |
|  | 28 | cymbalta 30mg gastro-resistant capsule | 1141201834 | N06AX21 | Duloxetine | Anticholinergic |
|  | 29 | anafranil 10mg capsule | 1140867690 | N06AA04 | Clomipramine | Anticholinergic |
|  | 30 | doxepin | 1140867640 | N06AA12 | Doxepin | Anticholinergic |
|  | 31 | moclobemide | 1140867920 | N06AG02 | Moclobemide | Anticholinergic |
|  | 32 | yentreve 20mg gastro-resistant capsule | 1141200570 | N06AX21 | Duloxetine | Anticholinergic |
|  | 33 | phenelzine | 1140867850 | N06AF03 | Phenelzine | Non-anticholinergic |
|  | 34 | fluvoxamine | 1140879544 | N06AB08 | Fluvoxamine | Non-anticholinergic |
|  | 35 | oxactin 20mg capsule | 1141174756 | N06AB03 | Fluoxetine | Anticholinergic |
|  | 36 | surmontil 10mg tablet | 1140867758 | N06AA06 | Trimipramine | Anticholinergic |
|  | 37 | tryptophan product | 1140867960 | N06AX02 | Tryptophan | Non-anticholinergic |
|  | 38 | zyban 150mg m/r tablet | 1141176858 | N06AX12 | Bupropion | Non-anticholinergic |
|  | 39 | tranylcypromine | 1140867914 | N06AF04 | Tranylcypromine | Non-anticholinergic |
|  | 40 | bupropion | 1141176854 | N06AX12 | Bupropion | Non-anticholinergic |
|  | 41 | allegron 10mg tablet | 1140867820 | N06AA10 | Nortriptyline | Anticholinergic |

**References:**

Coupland CAC, Hill T, Dening T, Morriss R, Moore M, Hippisley-Cox J. Anticholinergic Drug Exposure and the Risk of Dementia: A Nested Case-Control Study. JAMA Internal Medicine. 2019;179(8):1084-93

Richardson K, Fox C, Maidment I, Steel N, Loke YK, Arthur A, et al. Anticholinergic drugs and risk of dementia: case-control study. BMJ. 2018;361:k1315.

Lavrador M, Cabral AC, Veríssimo MT, Fernandez-Llimos F, Figueiredo IV, Castel-Branco MM. A Universal Pharmacological-Based List of Drugs with Anticholinergic Activity. Pharmaceutics. 2023;15(1).

# Supplementary Table 4. Baseline characteristics of all eligible participants and those with completed baseline data included in the main analysis.

| **Variable^a^** | **Eligible participants** | **Participants with completed baseline data** |
| --- | --- | --- |
| **N (%)** | 502157 | 461464 |
| **Age, years** | 56.53 (8.09) | 57 (8.1) |
| **Male** | 228942 (45.6) | 211520 (45.8) |
| **Ethnicity** |  |  |
| White | 472377 (94.1) | 439517 (95.2) |
| Others | 27005 (5.4) | 21947 (4.8) |
| Missing | 2775 (0.6) | - |
| **Socioeconomic status** |  |  |
| Low | 101279 (20.2) | 95466 (20.7) |
| Intermediate | 300127 (59.8) | 278107 (60.3) |
| High | 100125 (19.9) | 87891 (19.0) |
| Missing | 626 (0.1) | - |
| **Less education** | 310355 (61.8) | 281077 (60.9) |
| **Living alone** | 92842 (18.5) | 84344 (18.3) |
| **Excessive alcohol consumption** | 115134 (22.9) | 107817 (23.4) |
| **Current smoking** | 52944 (10.5) | 47560 (10.3) |
| **Physical inactivity** | 76142 (15.2) | 71015 (15.4) |
| **Sleep duration, hour** |  |  |
| < 7 | 123169 (24.5) | 111953 (24.3) |
| 7-8 | 192286 (38.3) | 179608 (38.9) |
| >=8 | 182493 (36.3) | 169903 (36.8) |
| Missing | 4209 (0.8) | - |
| **Social isolation** | 71807 (14.3) | 64856 (14.1) |
| **Overall health** |  |  |
| Poor | 22723 (4.5) | 19739 (4.3) |
| Fair | 105254 (21.0) | 93095 (20.2) |
| Good | 288875 (57.5) | 269621 (58.4) |
| Excellent | 81823 (16.3) | 79009 (17.1) |
| Missing | 3482 (0.7) | - |
| **Hyperlipidemia** | 74828 (14.9) | 67876 (14.7) |
| **Diabetes** | 147314 (29.3) | 133823 (29.0) |
| **Hypertension** | 280469 (55.9) | 257119 (55.7) |
| **Traumatic brain injury** | 7856 (1.6) | 6987 (1.5) |
| **Hearing loss** | 134920 (26.9) | 123309 (26.7) |
| **Vision loss** | 43153 (8.6) | 39188 (8.5) |
| **Obesity** | 122256 (24.3) | 110699 (24.0) |
| **Depression** | 41863 (8.3) | 37575 (8.1) |
| **PHQ-2 total score** | 0.58 (1.12) | 0.58 (1.11) |
| **Anxiety** | 27627 (5.5) | 24820 (5.4) |
| **With chronic pain** | 218498 (43.5) | 197938 (42.9) |
| **Insomnia** | 141295 (28.1) | 127509 (27.6) |
| **Using other Anticholinergics** | 25411 (5.1) | 22584 (4.9) |

^a^ Values are mean (SD) for age and PHQ-2 total score and N (%) for others.

# Supplementary Table 5. Number of incident dementia cases included in this study.

| **Dementia Category** | **ICD-10^*^** | **Eligible participants**  **N= 502,157 ^a^** | **Participants with completed baseline data**  **N= 461,464 ^b^** |
| --- | --- | --- | --- |
| Any Dementia | F00-03, G30 | 9,045 | 7,922 |
| Alzheimer’s disease | F00, G30 | 4,277 | 3,777 |
| Vascular dementia | F01 | 2,150 | 1,859 |

^a^ The mean (SD) of follow-up duration was 13.4 (2.1) years.

^b^ The mean (SD) of follow-up duration was 13.4 (2.1) years.

# Supplementary Table 6. Subgroup analysis for the association between antidepressant use and dementia across clinical indication subgroups

| **Subgroups** | **N** | **Dementia case  N (%)** | **HR (95% CI) of antidepressants use ^a^** | **p** |
| --- | --- | --- | --- | --- |
| Diagnosed depression |  |  |  |  |
| No | 423889 | 7053 (1.7) | **1.55 [1.40, 1.71]** | **<0.001** |
| Yes | 37575 | 869 (2.3) | **1.38 [1.20, 1.59]** | **<0.001** |
| Baseline PHQ-2 score |  |  |  |  |
| ≤2 | 382100 | 6441 (1.7) | **1.62 [1.48, 1.77]** | **<0.001** |
| >2 | 79364 | 1481 (1.9) | **1.53 [1.35, 1.72]** | **<0.001** |
| Anxiety |  |  |  |  |
| No | 436644 | 7413 (1.7) | **1.47 [1.35, 1.61]** | **<0.001** |
| Yes | 24820 | 509 (2.1) | **1.41 [1.16, 1.72]** | **0.001** |
| Chronic pain |  |  |  |  |
| No | 263526 | 3946 (1.5) | **1.58 [1.37, 1.83]** | **<0.001** |
| Yes | 197938 | 3976 (2.0) | **1.42 [1.29, 1.57]** | **<0.001** |
| Insomnia |  |  |  |  |
| No | 333955 | 5577 (1.7) | **1.58 [1.42, 1.75]** | **<0.001** |
| Yes | 127509 | 2345 (1.8) | **1.31 [1.15, 1.50]** | **<0.001** |

**Note**: Bold: *p*<0.05

**^a^** Model was adjusted for age, sex, ethnicity, socioeconomic status, less education, living alone status, excessive alcohol consumption, current smoking status, physical inactivity, sleeping duration categories, social isolation, overall health, hyperlipidemia, diabetes, hypertension, traumatic brain injury, hearing loss, vision loss, obesity, and using other anticholinergics, while depression, PHQ-2 total score, anxiety, self-reported chronic pain, and self-reported insomnia were adjusted when appropriate.

# Supplementary Table 7. Subgroup analysis for the association between antidepressant use and dementia across baseline demographic factors.

| **Subgroups** | **N** | **Dementia case (%)** | **HR (95% CI) of antidepressants use ^a^** | **p** | **P for interact** |
| --- | --- | --- | --- | --- | --- |
| Age |  |  |  |  | **0.002** |
| <60 | 261004 | 898 (0.3) | **1.49 [1.19, 1.86]** | **0.001** |  |
| ≥60 | 200460 | 7024 (3.5) | **1.43 [1.31, 1.57]** | **<0.001** |  |
| sex |  |  |  |  | 0.107 |
| Female | 249944 | 3750 (1.5) | **1.37 [1.23, 1.53]** | **<0.001** |  |
| Male | 211520 | 4172 (2.0) | **1.62 [1.43, 1.84]** | **<0.001** |  |
| Ethnicity |  |  |  |  | 0.766 |
| White | 439517 | 7642 (1.7) | **1.49 [1.37, 1.61]** | **<0.001** |  |
| Others | 21947 | 280 (1.3) | 1.06 [0.65, 1.74] | 0.813 |  |
| Socioeconomic status |  |  |  |  | 0.785 |
| Low | 95466 | 1556 (1.6) | **1.48 [1.21, 1.80]** | **<0.001** |  |
| Intermediate | 278107 | 4555 (1.6) | **1.51 [1.35, 1.68]** | **<0.001** |  |
| High | 87891 | 1811 (2.1) | **1.39 [1.19, 1.62]** | **<0.001** |  |
| Less education |  |  |  |  | **0.008** |
| No | 180387 | 2083 (1.2) | **1.67 [1.41, 1.97]** | **<0.001** |  |
| Yes | 281077 | 5839 (2.1) | **1.41 [1.29, 1.55]** | **<0.001** |  |
| Living alone |  |  |  |  | **0.013** |
| No | 377120 | 5951 (1.6) | **1.54 [1.40, 1.69]** | **<0.001** |  |
| Yes | 84344 | 1971 (2.3) | **1.31 [1.12, 1.53]** | **0.001** |  |

**Note**: Bold: *p*<0.05

**^a^** Model was adjusted for age, sex, ethnicity, socioeconomic status, less education, and living alone when appropriate and adjusted for excessive alcohol consumption, current smoking status, physical inactivity, sleeping duration categories, social isolation, overall health, hyperlipidemia, diabetes, hypertension, traumatic brain injury, hearing loss, vision loss, obesity, depression, PHQ-2 total score, anxiety, self-reported chronic pain, self-reported insomnia, and using other anticholinergics.

# Supplementary Table 8. Association of antidepressant use with normalized neuroimaging outcomes (z-score).

| **Neuroimaging outcome** | **Non-antidepressant use** | **Antidepressant use** | **p** |
| --- | --- | --- | --- |
| **Number of participants** | 40095 | 2181 |  |
| **Volume of brain, grey+white matter** | |  |  |
| Mean (SD) | 0.00 (1.00) | 0.03 (0.98) |  |
| β (95% CI) |  |  |  |
| Model 1 ^a^ | Ref | **-0.054 [-0.090, -0.019]** | **0.003** |
| Model 2 ^b^ | Ref | **-0.051 [-0.086, -0.015]** | **0.005** |
| Model 3 ^c^ | Ref | **-0.038 [-0.074, -0.002]** | **0.040** |
| Model 4 ^d^ | Ref | **-0.058 [-0.099, -0.018]** | **0.005** |
| Model 5 ^e^ | Ref | **-0.058 [-0.099, -0.018]** | **0.005** |
| **Volume of white matter** |  |  |  |
| Mean (SD) | 0.00 (1.00) | 0.00 (0.98) |  |
| β (95% CI) |  |  |  |
| Model 1 ^a^ | Ref | 0.021 [-0.020, 0.061] | 0.326 |
| Model 2 ^b^ | Ref | 0.022 [-0.019, 0.063] | 0.292 |
| Model 3 ^c^ | Ref | 0.022 [-0.020, 0.063] | 0.310 |
| Model 4 ^d^ | Ref | -0.007 [-0.054, 0.040] | 0.770 |
| Model 5 ^e^ | Ref | -0.008 [-0.055, 0.039] | 0.741 |
| **Volume of grey matter** |  |  |  |
| Mean (SD) | 0.00 (1.00) | 0.04 (0.99) |  |
| β (95% CI) |  |  |  |
| Model 1 ^a^ | Ref | **-0.101 [-0.133, -0.068]** | **<0.001** |
| Model 2 ^b^ | Ref | **-0.096 [-0.128, -0.064]** | **<0.001** |
| Model 3 ^c^ | Ref | **-0.076 [-0.109, -0.043]** | **<0.001** |
| Model 4 ^d^ | Ref | **-0.083 [-0.120, -0.046]** | **<0.001** |
| Model 5 ^e^ | Ref | **-0.082 [-0.119, -0.045]** | **<0.001** |
| **Total volume of white matter hyperintensities** | | |  |
| Mean (SD) | -0.01 (0.99) | 0.11 (1.17) |  |
| β (95% CI) |  |  |  |
| Model 1 ^a^ | Ref | **0.138 [0.098, 0.178]** | **<0.001** |
| Model 2 ^b^ | Ref | **0.130 [0.090, 0.171]** | **<0.001** |
| Model 3 ^c^ | Ref | **0.101 [0.061, 0.142]** | **<0.001** |
| Model 4 ^d^ | Ref | **0.131 [0.085, 0.176]** | **<0.001** |
| Model 5 ^e^ | Ref | **0.131 [0.085, 0.177]** | **<0.001** |
| **Volume of hippocampus** |  |  |  |
| Mean (SD) | 0.00 (1.00) | 0.07 (0.97) |  |
| β (95% CI) |  |  |  |
| Model 1 ^a^ | Ref | -0.035 [-0.074, 0.005] | 0.086 |
| Model 2 ^b^ | Ref | -0.028 [-0.068, 0.011] | 0.159 |
| Model 3 ^c^ | Ref | -0.019 [-0.060, 0.021] | 0.345 |
| Model 4 ^d^ | Ref | -0.044 [-0.090, 0.001] | 0.056 |
| Model 5 ^e^ | Ref | -0.044 [-0.089, 0.002] | 0.060 |
| **Volume of grey matter in Hippocampus** | |  |  |
| Mean (SD) | 0.00 (1.00) | 0.03 (1.03) |  |
| β (95% CI) |  |  |  |
| Model 1 ^a^ | Ref | **-0.078 [-0.117, -0.038]** | **<0.001** |
| Model 2 ^b^ | Ref | **-0.073 [-0.112, -0.033]** | **<0.001** |
| Model 3 ^c^ | Ref | **-0.067 [-0.107, -0.027]** | **0.001** |
| Model 4 ^d^ | Ref | **-0.078 [-0.124, -0.033]** | **0.001** |
| Model 5 ^e^ | Ref | **-0.079 [-0.124, -0.033]** | **0.001** |
| **Volume of Cerebellum-Cortex** | |  |  |
| Mean (SD) | 0.00 (1.00) | -0.03 (0.99) |  |
| β (95% CI) |  |  |  |
| Model 1 ^a^ | Ref | **-0.076 [-0.118, -0.034]** | **<0.001** |
| Model 2 ^b^ | Ref | **-0.068 [-0.110, -0.026]** | **0.002** |
| Model 3 ^c^ | Ref | **-0.050 [-0.092, -0.007]** | **0.024** |
| Model 4 ^d^ | Ref | -0.033 [-0.081, 0.016] | 0.189 |
| Model 5 ^e^ | Ref | -0.033 [-0.082, 0.015] | 0.181 |
| **Volume of Basal-nucleus** | |  |  |
| Mean (SD) | 0.00 (1.00) | -0.04 (0.98) |  |
| β (95% CI) |  |  |  |
| Model 1 ^a^ | Ref | **-0.101 [-0.142, -0.061]** | **<0.001** |
| Model 2 ^b^ | Ref | **-0.096 [-0.137, -0.056]** | **<0.001** |
| Model 3 ^c^ | Ref | **-0.100 [-0.141, -0.059]** | **<0.001** |
| Model 4 ^d^ | Ref | **-0.115 [-0.161, -0.069]** | **<0.001** |
| Model 5 ^e^ | Ref | **-0.115 [-0.162, -0.069]** | **<0.001** |
| **Volume of lateral orbitofrontal** | |  |  |
| Mean (SD) | 0.00 (1.00) | 0.02 (0.99) |  |
| β (95% CI) |  |  |  |
| Model 1 ^a^ | Ref | **-0.064 [-0.104, -0.023]** | **0.002** |
| Model 2 ^b^ | Ref | **-0.061 [-0.102, -0.020]** | **0.003** |
| Model 3 ^c^ | Ref | **-0.042 [-0.084, -0.001]** | **0.044** |
| Model 4 ^d^ | Ref | **-0.051 [-0.098, -0.004]** | **0.032** |
| Model 5 ^e^ | Ref | **-0.050 [-0.097, -0.003]** | **0.036** |

**Note:** Bolded values: *p*<0.05; *P* values shown in this table are unadjusted. Bonferroni-corrected significance for the primary neuroimaging analysis is indicated in Figure 2 based on Model 5.

^a^ Model 1: Baseline demographic factors: age, sex, ethnicity, socioeconomic status, less education, and living alone status;

^b^ Model 2: Model 1+lifestyle factors: excessive alcohol consumption, current smoking status, physical inactivity, sleeping duration categories, and social isolation;

^c^ Model 3: Model 2+health indicators: overall health, hyperlipidemia, diabetes, hypertension, traumatic brain injury, hearing loss, vision loss, and obesity;

^d^ Model 4: Model 3+ antidepressant indication factors: depression, PHQ-2 total score, anxiety, self-reported chronic pain, and self-reported insomnia;

^e^ Model 5: Model 4+ using other anticholinergics

# Supplementary Table 9. Association of antidepressant use with normalized neuroimaging outcomes.

| **Neuroimaging outcomes** | **Non-antidepressant use** | **Antidepressant use** | **p** |
| --- | --- | --- | --- |
| **Number of participants** | 40095 | 2181 |  |
| **Volume of brain, grey+white matter** | |  |  |
| Mean (SD) | 1490273.17 (73844.82) | 1492574.61 (72457.72) |  |
| β (95% CI) |  |  |  |
| Model 1 ^a^ | Ref | **-4017.84 [-6634.60, -1401.08]** | **0.003** |
| Model 2 ^b^ | Ref | **-3733.85 [-6345.26, -1122.43]** | **0.005** |
| Model 3 ^c^ | Ref | **-2782.47 [-5432.04, -132.90]** | **0.040** |
| Model 4 ^d^ | Ref | **-4310.27 [-7302.64, -1317.91]** | **0.005** |
| Model 5 ^e^ | Ref | **-4287.18 [-7283.11, -1291.25]** | **0.005** |
| **Volume of white matter** | |  |  |
| Mean (SD) | 699888.77 (40826.04) | 700082.74 (39947.71) |  |
| β (95% CI) |  |  |  |
| Model 1 ^a^ | Ref | 837.12 [-833.64, 2507.88] | 0.326 |
| Model 2 ^b^ | Ref | 900.19 [-772.83, 2573.21] | 0.292 |
| Model 3 ^c^ | Ref | 880.95 [-819.14, 2581.03] | 0.310 |
| Model 4 ^d^ | Ref | -286.56 [-2206.76, 1633.65] | 0.770 |
| Model 5 ^e^ | Ref | -323.64 [-2246.12, 1598.84] | 0.741 |
| **Volume of grey matter** | |  |  |
| Mean (SD) | 790384.41 (48301.43) | 792491.77 (47910.71) |  |
| β (95% CI) |  |  |  |
| Model 1 ^a^ | Ref | **-4855.09 [-6423.59, -3286.58]** | **<0.001** |
| Model 2 ^b^ | Ref | **-4634.16 [-6196.88, -3071.44]** | **<0.001** |
| Model 3 ^c^ | Ref | **-3663.51 [-5242.42, -2084.61]** | **<0.001** |
| Model 4 ^d^ | Ref | **-4023.82 [-5807.02, -2240.62]** | **<0.001** |
| Model 5 ^e^ | Ref | **-3963.65 [-5748.94, -2178.36]** | **<0.001** |
| **Total volume of white matter hyperintensities** | | |  |
| Mean (SD) |  |  |  |
| β (95% CI) | 6548.77 (8406.75) | 7527.75 (9901.85) |  |
| Model 1 ^a^ | Ref | **1170.47 [828.29, 1512.65]** | **<0.001** |
| Model 2 ^b^ | Ref | **1107.94 [765.25, 1450.63]** | **<0.001** |
| Model 3 ^c^ | Ref | **861.68 [516.67, 1206.68]** | **<0.001** |
| Model 4 ^d^ | Ref | **1108.48 [718.79, 1498.17]** | **<0.001** |
| Model 5 ^e^ | Ref | **1111.30 [721.15, 1501.46]** | **<0.001** |
| **Volume of hippocampus** | |  |  |
| Mean (SD) | 9869.24 (1158.35) | 9958.77 (1124.92) |  |
| β (95% CI) |  |  |  |
| Model 1 ^a^ | Ref | -40.06 [-85.83, 5.70] | 0.086 |
| Model 2 ^b^ | Ref | -32.91 [-78.74, 12.91] | 0.159 |
| Model 3 ^c^ | Ref | -22.44 [-68.98, 24.09] | 0.345 |
| Model 4 ^d^ | Ref | -51.27 [-103.81, 1.28] | 0.056 |
| Model 5 ^e^ | Ref | -50.41 [-103.02, 2.20] | 0.060 |
| **Volume of grey matter in Hippocampus** | |  |  |
| Mean (SD) | 11017.39 (979.75) | 11052.36 (1006.54) |  |
| β (95% CI) |  |  |  |
| Model 1 ^a^ | Ref | **-76.20 [-114.92, -37.48]** | **<0.001** |
| Model 2 ^b^ | Ref | **-71.19 [-109.97, -32.41]** | **<0.001** |
| Model 3 ^c^ | Ref | **-65.72 [-105.08, -26.36]** | **0.001** |
| Model 4 ^d^ | Ref | **-76.91 [-121.36, -32.46]** | **0.001** |
| Model 5 ^e^ | Ref | **-77.25 [-121.76, -32.75]** | **0.001** |
| **Volume of Cerebellum-Cortex** | |  |  |
| Mean (SD) | 144321.13 (12514.88) | 143892.27 (12338.12) |  |
| β (95% CI) |  |  |  |
| Model 1 ^a^ | Ref | **-949.38 [-1478.35, -420.41]** | **<0.001** |
| Model 2 ^b^ | Ref | **-850.75 [-1380.52, -320.98]** | **0.002** |
| Model 3 ^c^ | Ref | **-619.43 [-1156.54, -82.31]** | **0.024** |
| Model 4 ^d^ | Ref | -406.50 [-1013.22, 200.21] | 0.189 |
| Model 5 ^e^ | Ref | -414.45 [-1021.89, 192.99] | 0.181 |
| **Volume of Basal-nucleus** | |  |  |
| Mean (SD) | 1091.80 (106.50) | 1086.90 (104.08) |  |
| β (95% CI) |  |  |  |
| Model 1 ^a^ | Ref | **-10.77 [-15.06, -6.48]** | **<0.001** |
| Model 2 ^b^ | Ref | **-10.24 [-14.54, -5.95]** | **<0.001** |
| Model 3 ^c^ | Ref | **-10.64 [-15.00, -6.27]** | **<0.001** |
| Model 4 ^d^ | Ref | **-12.22 [-17.15, -7.30]** | **<0.001** |
| Model 5 ^e^ | Ref | **-12.27 [-17.21, -7.34]** | **<0.001** |
| **Volume of lateral orbitofrontal** | |  |  |
| Mean (SD) | 20742.20 (1628.37) | 20773.28 (1607.71) |  |
| β (95% CI) |  |  |  |
| Model 1 ^a^ | Ref | **-103.60 [-169.81, -37.40]** | **0.002** |
| Model 2 ^b^ | Ref | **-99.11 [-165.42, -32.81]** | **0.003** |
| Model 3 ^c^ | Ref | **-69.15 [-136.38, -1.91]** | **0.044** |
| Model 4 ^d^ | Ref | **-82.95 [-158.90, -7.00]** | **0.032** |
| Model 5 ^e^ | Ref | **-81.32 [-157.36, -5.28]** | **0.036** |

**Note:** Bolded values: *p*<0.05;

^a^ Model 1: Baseline demographic factors: age, sex, ethnicity, socioeconomic status, less education, and living alone status;

^b^ Model 2: Model 1+lifestyle factors: excessive alcohol consumption, current smoking status, physical inactivity, sleeping duration categories, and social isolation;

^c^ Model 3: Model 2+health indicators: overall health, hyperlipidemia, diabetes, hypertension, traumatic brain injury, hearing loss, vision loss, and obesity;

^d^ Model 4: Model 3+ antidepressant indication factors: depression, PHQ-2 total score, anxiety, self-reported chronic pain, and self-reported insomnia;

^e^ Model 5: Model 4+ using other anticholinergics.

# Supplementary Table 10. Association of antidepressant use with crude neuroimaging outcomes.

| **Neuroimaging outcomes** | Non-antidepressant use | Antidepressant use | p |
| --- | --- | --- | --- |
| **Number of participants** | 40095 | 2181 |  |
| **Volume of brain, grey+white matter** | |  |  |
| Mean (SD) | 1158571.39 (111554.95) | 1137776.88 (108040.76) |  |
| β (95% CI) |  |  |  |
| Model 1 ^a^ | Ref | -820.13 [-4631.77, 2991.52] | 0.673 |
| Model 2 ^b^ | Ref | -312.87 [-4131.52, 3505.78] | 0.872 |
| Model 3 ^c^ | Ref | 1943.62 [-1931.64, 5818.89] | 0.326 |
| Model 4 ^d^ | Ref | 816.19 [-3560.63, 5193.02] | 0.715 |
| Model 5 ^e^ | Ref | 1065.07 [-3316.70, 5446.84] | 0.634 |
| **Volume of white matter** |  |  |  |
| Mean (SD) | 544799.17 (61618.32) | 534304.00 (59398.56) |  |
| β (95% CI) |  |  |  |
| Model 1 ^a^ | Ref | 1712.10 [-429.19, 3853.38] | 0.117 |
| Model 2 ^b^ | Ref | 1890.68 [-255.64, 4037.01] | 0.084 |
| Model 3 ^c^ | Ref | **2590.18 [411.74, 4768.62]** | **0.020** |
| Model 4 ^d^ | Ref | 1665.38 [-794.98, 4125.74] | 0.185 |
| Model 5 ^e^ | Ref | 1747.79 [-715.45, 4211.04] | 0.164 |
| **Volume of grey matter** |  |  |  |
| Mean (SD) | 613772.24 (55872.07) | 603472.81 (54552.53) |  |
| β (95% CI) |  |  |  |
| Model 1 ^a^ | Ref | **-2532.31 [-4465.74, -598.89]** | **0.010** |
| Model 2 ^b^ | Ref | **-2203.65 [-4138.91, -268.38]** | **0.026** |
| Model 3 ^c^ | Ref | -646.63 [-2608.38, 1315.11] | 0.518 |
| Model 4 ^d^ | Ref | -849.27 [-3065.01, 1366.47] | 0.453 |
| Model 5 ^e^ | Ref | -682.81 [-2900.96, 1535.33] | 0.546 |
| **Total volume of white matter hyperintensities** | |  |  |
| Mean (SD) | 5141.65 (6716.70) | 5758.84 (7576.11) |  |
| β (95% CI) |  |  |  |
| Model 1 ^a^ | Ref | **883.24 [610.51, 1155.97]** | **<0.001** |
| Model 2 ^b^ | Ref | **834.44 [561.29, 1107.59]** | **<0.001** |
| Model 3 ^c^ | Ref | **643.66 [368.59, 918.73]** | **<0.001** |
| Model 4 ^d^ | Ref | **835.94 [525.25, 1146.64]** | **<0.001** |
| Model 5 ^e^ | Ref | **840.82 [529.75, 1151.88]** | **<0.001** |
| **Volume of hippocampus** | |  |  |
| Mean (SD) | 7644.59 (893.27) | 7566.97 (862.74) |  |
| β (95% CI) |  |  |  |
| Model 1 ^a^ | Ref | -14.86 [-50.45, 20.73] | 0.413 |
| Model 2 ^b^ | Ref | -7.39 [-43.03, 28.26] | 0.685 |
| Model 3 ^c^ | Ref | 10.26 [-25.93, 46.46] | 0.578 |
| Model 4 ^d^ | Ref | -11.80 [-52.68, 29.08] | 0.572 |
| Model 5 ^e^ | Ref | -9.71 [-50.64, 31.22] | 0.642 |
| **Volume of grey matter in Hippocampus** | |  |  |
| Mean (SD) | 8542.87 (833.30) | 8403.75 (832.59) |  |
| β (95% CI) |  |  |  |
| Model 1 ^a^ | Ref | **-42.28 [-74.58, -9.97]** | **0.010** |
| Model 2 ^b^ | Ref | **-36.34 [-68.71, -3.97]** | **0.028** |
| Model 3 ^c^ | Ref | -21.36 [-54.23, 11.51] | 0.203 |
| Model 4 ^d^ | Ref | -27.88 [-65.00, 9.24] | 0.141 |
| Model 5 ^e^ | Ref | -26.40 [-63.57, 10.77] | 0.164 |
| **Volume of Cerebellum-Cortex** | |  |  |
| Mean (SD) | 112018.05 (11831.00) | 109489.55 (11178.67) |  |
| β (95% CI) |  |  |  |
| Model 1 ^a^ | Ref | **-526.06 [-958.18, -93.94]** | **0.017** |
| Model 2 ^b^ | Ref | -423.66 [-856.58, 9.26] | 0.055 |
| Model 3 ^c^ | Ref | -103.70 [-543.08, 335.69] | 0.644 |
| Model 4 ^d^ | Ref | 71.44 [-424.76, 567.64] | 0.778 |
| Model 5 ^e^ | Ref | 87.81 [-408.97, 584.59] | 0.729 |
| **Volume of Basal-nucleus** | |  |  |
| Mean (SD) | 847.74 (100.18) | 827.56 (96.76) |  |
| β (95% CI) |  |  |  |
| Model 1 ^a^ | Ref | **-6.55 [-10.29, -2.81]** | **0.001** |
| Model 2 ^b^ | Ref | **-5.94 [-9.68, -2.19]** | **0.002** |
| Model 3 ^c^ | Ref | **-5.14 [-8.94, -1.34]** | **0.008** |
| Model 4 ^d^ | Ref | **-6.34 [-10.63, -2.04]** | **0.004** |
| Model 5 ^e^ | Ref | **-6.20 [-10.50, -1.90]** | **0.005** |
| **Volume of lateral orbitofrontal** | |  |  |
| Mean (SD) | 16109.73 (1692.54) | 15822.76 (1665.57) |  |
| β (95% CI) |  |  |  |
| Model 1 ^a^ | Ref | -46.13 [-110.06, 17.81] | 0.157 |
| Model 2 ^b^ | Ref | -38.82 [-102.88, 25.24] | 0.235 |
| Model 3 ^c^ | Ref | 4.52 [-60.46, 69.49] | 0.892 |
| Model 4 ^d^ | Ref | -3.66 [-77.05, 69.73] | 0.922 |
| Model 5 ^e^ | Ref | 0.68 [-72.79, 74.15] | 0.986 |

**Note:** Bolded values: *p*<0.05;

^a^ Model 1: Baseline demographic factors: age, sex, ethnicity, socioeconomic status, less education, and living alone status;

^b^ Model 2: Model 1+lifestyle factors: excessive alcohol consumption, current smoking status, physical inactivity, sleeping duration categories, and social isolation;

^c^ Model 3: Model 2+health indicators: overall health, hyperlipidemia, diabetes, hypertension, traumatic brain injury, hearing loss, vision loss, and obesity;

^d^ Model 4: Model 3+ antidepressant indication factors: depression, PHQ-2 total score, anxiety, self-reported chronic pain, and self-reported insomnia;

^e^ Model 5: Model 4+ using other anticholinergics.

# Supplementary Table 11. Risk of dementia associated with antidepressants in different anticholinergic activity.

| **Anticholinergic activity criteria** | **All-cause dementia** | **Non-antidepressant use** | **Antidepressant use** | | | |
| --- | --- | --- | --- | --- | --- | --- |
|  |  |  | **Non-anticholinergic** | | **Anticholinergic** | |
| (Coupland et al., 2019) | N | 427743 | 20028 |  | 12230 |  |
|  | Number (%) of dementia event | 6904 (1.6) | 558 (2.8) |  | 405 (3.3) |  |
|  | Model 1 ^a^ | Ref | **2.33 [2.14, 2.54]** | **<0.001** | **1.83 [1.65, 2.02]** | **<0.001** |
|  | Model 2 ^b^ | Ref | **2.27 [2.08, 2.48]** | **<0.001** | **1.77 [1.60, 1.96]** | **<0.001** |
|  | Model 3 ^c^ | Ref | **1.88 [1.72, 2.06]** | **<0.001** | **1.42 [1.29, 1.58]** | **<0.001** |
|  | Model 4 ^d^ | Ref | **1.66 [1.49, 1.85]** | **<0.001** | **1.36 [1.22, 1.51]** | **<0.001** |
|  | Model 5 ^e^ | Ref | **1.63 [1.47, 1.82]** | **<0.001** | **1.33 [1.19, 1.47]** | **<0.001** |
| (Richardson et al., 2018) | N | 427743 | 12819 |  | 19439 |  |
|  | Number (%) of dementia event | 6904 (1.6) | 348 (2.7) |  | 615 (3.2) |  |
|  | Model 1 ^a^ | Ref | **2.25 [2.02, 2.51]** | **<0.001** | **2.01 [1.85, 2.18]** | **<0.001** |
|  | Model 2 ^b^ | Ref | **2.19 [1.96, 2.44]** | **<0.001** | **1.95 [1.80, 2.12]** | **<0.001** |
|  | Model 3 ^c^ | Ref | **1.80 [1.61, 2.01]** | **<0.001** | **1.59 [1.46, 1.73]** | **<0.001** |
|  | Model 4 ^d^ | Ref | **1.56 [1.37, 1.76]** | **<0.001** | **1.47 [1.34, 1.61]** | **<0.001** |
|  | Model 5 ^e^ | Ref | **1.52 [1.35, 1.73]** | **<0.001** | **1.44 [1.31, 1.58]** | **<0.001** |
| (Lavrador et al., 2023) | N | 427743 | 3114 |  | 29144 |  |
|  | Number (%) of dementia event | 6904 (1.6) | 85 (2.7) |  | 878 (3.0) |  |
|  | Model 1 ^a^ | Ref | **2.20 [1.77, 2.72]** | **<0.001** | **2.08 [1.94, 2.23]** | **<0.001** |
|  | Model 2 ^b^ | Ref | **2.12 [1.71, 2.63]** | **<0.001** | **2.02 [1.89, 2.17]** | **<0.001** |
|  | Model 3 ^c^ | Ref | **1.81 [1.46, 2.24]** | **<0.001** | **1.64 [1.53, 1.77]** | **<0.001** |
|  | Model 4 ^d^ | Ref | **1.58 [1.26, 1.97]** | **<0.001** | **1.49 [1.37, 1.62]** | **<0.001** |
|  | Model 5 ^e^ | Ref | **1.54 [1.23, 1.92]** | **<0.001** | **1.46 [1.34, 1.58]** | **<0.001** |

**Note:** Bolded values: *p*<0.05;

^a^ Model 1: Baseline demographic factors: age, sex, ethnicity, socioeconomic status, less education, and living alone status;

^b^ Model 2: Model 1+lifestyle factors: excessive alcohol consumption, current smoking status, physical inactivity, sleeping duration categories, and social isolation;

^c^ Model 3: Model 2+health indicators: overall health, hyperlipidemia, diabetes, hypertension, traumatic brain injury, hearing loss, vision loss, and obesity;

^d^ Model 4: Model 3+ antidepressant indication factors: depression, PHQ-2 total score, anxiety, self-reported chronic pain, and self-reported insomnia;

^e^ Model 5: Model 4+ using other anticholinergics.

**References:**

Coupland CAC, Hill T, Dening T, Morriss R, Moore M, Hippisley-Cox J. Anticholinergic Drug Exposure and the Risk of Dementia: A Nested Case-Control Study. JAMA Internal Medicine. 2019;179(8):1084-93

Richardson K, Fox C, Maidment I, Steel N, Loke YK, Arthur A, et al. Anticholinergic drugs and risk of dementia: case-control study. BMJ. 2018;361:k1315.

Lavrador M, Cabral AC, Veríssimo MT, Fernandez-Llimos F, Figueiredo IV, Castel-Branco MM. A Universal Pharmacological-Based List of Drugs with Anticholinergic Activity. Pharmaceutics. 2023;15(1).

# Supplementary Table 12. Risk of dementia associated with antidepressants in different pharmacological subtypes.

| **All-cause dementia** | **Non-antidepressant use** | **Antidepressant use** | | | | | | | | | |
| --- | --- | --- | --- | --- | --- | --- | --- | --- | --- | --- | --- |
|  |  | **TCA** | | **SSRI** | | **NaSSA** | | **SNRIs** | | **Others** | |
| N | 427743 | 10297 | | 17455 | | 999 | | 2180 | | 1327 | |
| Number (%) of dementia event | 6904 (1.6) | 346 (3.4) | | 478 (2.7) | | 44 (4.4) | | 67 (3.1) | | 28 (2.1) | |
| Model 1 ^a^ | Ref | **1.81 [1.62, 2.01]** | **<0.001** | **2.26 [2.06, 2.48]** | **<0.001** | **3.17 [2.36, 4.27]** | **<0.001** | **2.46 [1.94, 3.13]** | **<0.001** | **1.66 [1.14, 2.40]** | **0.008** |
| Model 2 ^b^ | Ref | **1.76 [1.57, 1.96]** | **<0.001** | **2.21 [2.01, 2.42]** | **<0.001** | **3.03 [2.26, 4.08]** | **<0.001** | **2.37 [1.86, 3.02]** | **<0.001** | **1.62 [1.12, 2.35]** | **0.011** |
| Model 3 ^c^ | Ref | **1.40 [1.25, 1.56]** | **<0.001** | **1.83 [1.67, 2.01]** | **<0.001** | **2.41 [1.79, 3.25]** | **<0.001** | **1.95 [1.53, 2.48]** | **<0.001** | 1.44 [0.99, 2.08] | 0.056 |
| Model 4 ^d^ | Ref | **1.35 [1.21, 1.51]** | **<0.001** | **1.62 [1.45, 1.82]** | **<0.001** | **2.09 [1.54, 2.84]** | **<0.001** | **1.72 [1.34, 2.21]** | **<0.001** | 1.31 [0.90, 1.90] | 0.160 |
| Model 5 ^e^ | Ref | **1.31 [1.17, 1.47]** | **<0.001** | **1.60 [1.43, 1.80]** | **<0.001** | **2.05 [1.52, 2.79]** | **<0.001** | **1.67 [1.30, 2.15]** | **<0.001** | 1.27 [0.87, 1.85] | 0.209 |

**Note:** Bolded values: *p*<0.05;

^a^ Model 1: Baseline demographic factors: age, sex, ethnicity, socioeconomic status, less education, and living alone status;

^b^ Model 2: Model 1+lifestyle factors: excessive alcohol consumption, current smoking status, physical inactivity, sleeping duration categories, and social isolation;

^c^ Model 3: Model 2+health indicators: overall health, hyperlipidemia, diabetes, hypertension, traumatic brain injury, hearing loss, vision loss, and obesity;

^d^ Model 4: Model 3+ antidepressant indication factors: depression, PHQ-2 total score, anxiety, self-reported chronic pain, and self-reported insomnia;

^e^ Model 5: Model 4+ using other anticholinergics.

# Supplementary Table 13. Sensitivity analysis for association of antidepressant use with risk of dementia when excluding dementia case occurred in the first 5 years of follow-up.

|  | **Non-antidepressant use** | **Antidepressant use** | ***p*** |
| --- | --- | --- | --- |
| N | 427224 | 33605 |  |
| All-cause dementia |  |  |  |
| Number (%) of dementia event | 6385 (1.5) | 902 (2.7) |  |
| HR (95% CI) |  |  |  |
| Model 1 ^a^ | **Ref** | **2.04 [1.90, 2.19]** | **<0.001** |
| Model 2 ^b^ | **Ref** | **1.99 [1.86, 2.14]** | **<0.001** |
| Model 3 ^c^ | **Ref** | **1.63 [1.51, 1.75]** | **<0.001** |
| Model 4 ^d^ | **Ref** | **1.49 [1.37, 1.63]** | **<0.001** |
| Model 5 ^e^ | **Ref** | **1.46 [1.34, 1.59]** | **<0.001** |
| Alzheimer’s disease |  |  |  |
| Number (%) of Alzheimer’s disease event | 3057 (0.7) | 400 (1.2) |  |
| HR (95% CI) |  |  |  |
| Model 1 ^a^ | Ref | **1.84 [1.65, 2.04]** | **<0.001** |
| Model 2 ^b^ | Ref | **1.84 [1.66, 2.05]** | **<0.001** |
| Model 3 ^c^ | Ref | **1.62 [1.45, 1.80]** | **<0.001** |
| Model 4 ^d^ | Ref | **1.55 [1.37, 1.76]** | **<0.001** |
| Model 5 ^e^ | Ref | **1.52 [1.34, 1.73]** | **<0.001** |
| Vascular dementia |  |  |  |
| Number (%) of vascular dementia event | 1424 (0.3) | 233 (0.7) |  |
| HR (95% CI) |  |  |  |
| Model 1 ^a^ | Ref | **2.42 [2.10, 2.78]** | **<0.001** |
| Model 2 ^b^ | Ref | **2.31 [2.01, 2.66]** | **<0.001** |
| Model 3 ^c^ | Ref | **1.72 [1.49, 1.99]** | **<0.001** |
| Model 4 ^d^ | Ref | **1.45 [1.22, 1.72]** | **<0.001** |
| Model 5 ^e^ | Ref | **1.39 [1.17, 1.65]** | **<0.001** |

**Note:** Bolded values: *p*<0.05;

^a^ Model 1: Baseline demographic factors: age, sex, ethnicity, socioeconomic status, less education, and living alone status;

^b^ Model 2: Model 1+lifestyle factors: excessive alcohol consumption, current smoking status, physical inactivity, sleeping duration categories, and social isolation;

^c^ Model 3: Model 2+health indicators: overall health, hyperlipidemia, diabetes, hypertension, traumatic brain injury, hearing loss, vision loss, and obesity;

^d^ Model 4: Model 3+ antidepressant indication factors: depression, PHQ-2 total score, anxiety, self-reported chronic pain, and self-reported insomnia;

^e^ Model 5: Model 4+ using other anticholinergics.

# Supplementary Table 14. Sensitivity analysis for association of antidepressant use with risk of dementia among participants with cognitive function test in the baseline.

|  | **Non-antidepressant use** | **Antidepressant use** | ***p*** |
| --- | --- | --- | --- |
| N | 142552 | 10862 |  |
| **All-cause dementia** |  |  |  |
| Number (%) of dementia event | 1783 (1.3) | 252 (2.3) |  |
| HR (95% CI) |  |  |  |
| Model 1 ^a^ | Ref | **2.13 [1.87, 2.44]** | **<0.001** |
| Model 2 ^b^ | Ref | **2.08 [1.82, 2.38]** | **<0.001** |
| Model 3 ^c^ | Ref | **1.68 [1.47, 1.93]** | **<0.001** |
| Model 4 ^d^ | Ref | **1.49 [1.26, 1.76]** | **<0.001** |
| Model 5 ^e^ | Ref | **1.44 [1.22, 1.70]** | **<0.001** |
| Model 6 ^f^ | Ref | **1.42 [1.21, 1.68]** | **<0.001** |
| **Alzheimer’s disease** |  |  |  |
| Number (%) of Alzheimer’s disease event | 861 (0.6) | 118 (1.1) |  |
| HR (95% CI) |  |  |  |
| Model 1 ^a^ | Ref | **2.02 [1.67, 2.46]** | **<0.001** |
| Model 2 ^b^ | Ref | **2.01 [1.66, 2.45]** | **<0.001** |
| Model 3 ^c^ | Ref | **1.78 [1.46, 2.18]** | **<0.001** |
| Model 4 ^d^ | Ref | **1.61 [1.26, 2.05]** | **<0.001** |
| Model 5 ^e^ | Ref | **1.57 [1.23, 2.00]** | **<0.001** |
| Model 6 ^f^ | Ref | **1.54 [1.21, 1.96]** | **<0.001** |
| **Vascular dementia** |  |  |  |
| Number (%) of vascular dementia event | 385 (0.3) | 56 (0.5) |  |
| HR (95% CI) |  |  |  |
| Model 1 ^a^ | Ref | **2.21 [1.67, 2.94]** | **<0.001** |
| Model 2 ^b^ | Ref | **2.07 [1.55, 2.75]** | **<0.001** |
| Model 3 ^c^ | Ref | **1.44 [1.08, 1.94]** | **0.015** |
| Model 4 ^d^ | Ref | 1.17 [0.83, 1.66] | 0.370 |
| Model 5 ^e^ | Ref | 1.12 [0.79, 1.59] | 0.540 |
| Model 6 ^f^ | Ref | 1.11 [0.78, 1.58] | 0.555 |

**Note:** Bolded values: *p*<0.05;

^a^ Model 1: Baseline demographic factors: age, sex, ethnicity, socioeconomic status, less education, and living alone status;

^b^ Model 2: Model 1+lifestyle factors: excessive alcohol consumption, current smoking status, physical inactivity, sleeping duration categories, and social isolation;

^c^ Model 3: Model 2+health indicators: overall health, hyperlipidemia, diabetes, hypertension, traumatic brain injury, hearing loss, vision loss, and obesity;

^d^ Model 4: Model 3+ antidepressant indication factors: depression, PHQ-2 total score, anxiety, self-reported chronic pain, and self-reported insomnia;

^e^ Model 5: Model 4+ using other anticholinergics;

^f^ Model 6: Model 5+ fluid intelligence score and prospective memory.

# Supplementary Table 15. Sensitivity analysis for association of antidepressant use with risk of dementia when using competing risk regression model.

|  | **Non-antidepressant use** | **Antidepressant use** | ***p*** |
| --- | --- | --- | --- |
| N | 427743 | 33721 |  |
| All-cause dementia |  |  |  |
| Number (%) of dementia event | 6904 (1.6) | 1018 (3.0) |  |
| HR (95% CI) |  |  |  |
| Model 1 ^a^ | Ref | **2.02 [1.89, 2.16]** | **<0.001** |
| Model 2 ^b^ | Ref | **1.98 [1.85, 2.12]** | **<0.001** |
| Model 3 ^c^ | Ref | **1.65 [1.54, 1.77]** | **<0.001** |
| Model 4 ^d^ | Ref | **1.47 [1.35, 1.60]** | **<0.001** |
| Model 5 ^e^ | Ref | **1.45 [1.33, 1.57]** | **<0.001** |
| Alzheimer’s disease |  |  |  |
| Number (%) of Alzheimer’s disease event | 3320 (0.8) | 457 (1.4) |  |
| HR (95% CI) |  |  |  |
| Model 1 ^a^ | Ref | **1.83 [1.66, 2.02]** | **<0.001** |
| Model 2 ^b^ | Ref | **1.83 [1.66, 2.03]** | **<0.001** |
| Model 3 ^c^ | Ref | **1.66 [1.50, 1.84]** | **<0.001** |
| Model 4 ^d^ | Ref | **1.53 [1.35, 1.72]** | **<0.001** |
| Model 5 ^e^ | Ref | **1.51 [1.33, 1.70]** | **<0.001** |
| Vascular dementia |  |  |  |
| Number (%) of vascular dementia event | 1584 (0.4) | 275 (0.8) |  |
| HR (95% CI) |  |  |  |
| Model 1 ^a^ | Ref | **2.42 [2.12, 2.75]** | **<0.001** |
| Model 2 ^b^ | Ref | **2.31 [2.03, 2.64]** | **<0.001** |
| Model 3 ^c^ | Ref | **1.77 [1.54, 2.02]** | **<0.001** |
| Model 4 ^d^ | Ref | **1.46 [1.23, 1.72]** | **<0.001** |
| Model 5 ^e^ | Ref | **1.42 [1.20, 1.68]** | **<0.001** |

**Note:** Bolded values: *p*<0.05;

^a^ Model 1: Baseline demographic factors: age, sex, ethnicity, socioeconomic status, less education, and living alone status;

^b^ Model 2: Model 1+lifestyle factors: excessive alcohol consumption, current smoking status, physical inactivity, sleeping duration categories, and social isolation;

^c^ Model 3: Model 2+health indicators: overall health, hyperlipidemia, diabetes, hypertension, traumatic brain injury, hearing loss, vision loss, and obesity;

^d^ Model 4: Model 3+ antidepressant indication factors: depression, PHQ-2 total score, anxiety, self-reported chronic pain, and self-reported insomnia;

^e^ Model 5: Model 4+ using other anticholinergics.

# Supplementary Table 16. Sensitivity analysis for association of antidepressant use with risk of dementia with multiple imputations of missing data of covariates.

|  | Non-antidepressant use | Antidepressant use | *p* |
| --- | --- | --- | --- |
| N | 464485 | 37672 |  |
| All-cause dementia |  |  |  |
| Number (%) of dementia event | 7862 (1.7) | 1183 (3.1) |  |
| HR (95% CI) |  |  |  |
| Model 1 ^a^ | Ref | **2.10 [1.97, 2.23]** | **<0.001** |
| Model 2 ^b^ | Ref | **2.04 [1.91, 2.17]** | **<0.001** |
| Model 3 ^c^ | Ref | **1.65 [1.55, 1.76]** | **<0.001** |
| Model 4 ^d^ | Ref | **1.50 [1.39, 1.62]** | **<0.001** |
| Model 5 ^e^ | Ref | **1.47 [1.37, 1.59]** | **<0.001** |
| Alzheimer’s disease |  |  |  |
| Number (%) of Alzheimer’s disease event | 3755 (0.8) | 522 (1.4) |  |
| HR (95% CI) |  |  |  |
| Model 1 ^a^ | Ref | **1.88 [1.71, 2.06]** | **<0.001** |
| Model 2 ^b^ | Ref | **1.86 [1.70, 2.04]** | **<0.001** |
| Model 3 ^c^ | Ref | **1.64 [1.49, 1.81]** | **<0.001** |
| Model 4 ^d^ | Ref | **1.53 [1.37, 1.71]** | **<0.001** |
| Model 5 ^e^ | Ref | **1.51 [1.35, 1.69]** | **<0.001** |
| Vascular dementia |  |  |  |
| Number (%) of vascular dementia event | 1821 (0.4) | 329 (0.9) |  |
| HR (95% CI) |  |  |  |
| Model 1 ^a^ | Ref | **2.59 [2.30, 2.91]** | **<0.001** |
| Model 2 ^b^ | Ref | **2.46 [2.18, 2.77]** | **<0.001** |
| Model 3 ^c^ | Ref | **1.81 [1.60, 2.05]** | **<0.001** |
| Model 4 ^d^ | Ref | **1.54 [1.33, 1.79]** | **<0.001** |
| Model 5 ^e^ | Ref | **1.50 [1.29, 1.74]** | **<0.001** |

**Note:** Bolded values: *p*<0.05;

^a^ Model 1: Baseline demographic factors: age, sex, ethnicity, socioeconomic status, less education, and living alone status;

^b^ Model 2: Model 1+lifestyle factors: excessive alcohol consumption, current smoking status, physical inactivity, sleeping duration categories, and social isolation;

^c^ Model 3: Model 2+health indicators: overall health, hyperlipidemia, diabetes, hypertension, traumatic brain injury, hearing loss, vision loss, and obesity;

^d^ Model 4: Model 3+ antidepressant indication factors: depression, PHQ-2 total score, anxiety, self-reported chronic pain, and self-reported insomnia;

^e^ Model 5: Model 4+ using other anticholinergics.

# Supplementary Table 17. Baseline characteristics of propensity score matching (PSM) matched participants.

| **Variable** | **Non-antidepressants use** | **Antidepressants use** | **SMD** |
| --- | --- | --- | --- |
| **N (%)** | 33721 (50.0) | 33721 (50.0) |  |
| Age, years | 56.81 (7.9) | 56.45 (7.8) | 0.047 |
| Male | 10296 (30.5) | 10479 (31.1) | 0.012 |
| White | 32761 (97.2) | 32699 (97.0) | 0.011 |
| Less education | 22805 (67.6) | 23202 (68.8) | 0.025 |
| Socioeconomic status |  |  | 0.029 |
| Low | 5949 (17.6) | 5822 (17.3) |  |
| Intermediate | 19672 (58.3) | 19373 (57.5) |  |
| High | 8100 (24.0) | 8526 (25.3) |  |
| Living alone | 8142 (24.1) | 8438 (25.0) | 0.020 |
| Excessive alcohol consumption | 5819 (17.3) | 5972 (17.7) | 0.012 |
| Current smoking | 4976 (14.8) | 5457 (16.2) | 0.039 |
| Physical inactivity | 6831 (20.3) | 7328 (21.7) | 0.036 |
| Sleep duration, hour |  |  | 0.055 |
| < 7 | 9059 (26.9) | 8906 (26.4) |  |
| 7-8 | 10237 (30.4) | 9531 (28.3) |  |
| >=8 | 14425 (42.8) | 15284 (45.3) |  |
| Social isolation | 5885 (17.5) | 6319 (18.7) | 0.033 |
| Overall health |  |  | 0.094 |
| Poor | 4917 (14.6) | 5728 (17.0) |  |
| Fair | 11230 (33.3) | 11854 (35.2) |  |
| Good | 15392 (45.6) | 14315 (42.5) |  |
| Excellent | 2182 (6.5) | 1824 (5.4) |  |
| Hyperlipidemia | 6588 (19.5) | 6924 (20.5) | 0.025 |
| Diabetes | 12472 (37.0) | 12565 (37.3) | 0.006 |
| Hypertension | 19598 (58.1) | 19443 (57.7) | 0.009 |
| Traumatic brain injury | 773 (2.3) | 864 (2.6) | 0.018 |
| Hearing loss | 10671 (31.6) | 10948 (32.5) | 0.018 |
| Vision loss | 3194 (9.5) | 3286 (9.7) | 0.009 |
| Obesity | 10791 (32.0) | 11519 (34.2) | 0.046 |
| Depression | 16973 (50.3) | 19504 (57.8) | **0.151** |
| PHQ-2 total score | 1.27 (1.60) | 1.54 (1.71) | **0.164** |
| Anxiety | 6799 (20.2) | 7675 (22.8) | 0.063 |
| With chronic pain | 21439 (63.6) | 22063 (65.4) | 0.039 |
| Insomnia | 13925 (41.3) | 14375 (42.6) | 0.027 |
| Using other Anticholinergics | 5151 (15.3) | 5397 (16.0) | 0.020 |

# Supplementary Table 18. Sensitivity analysis for association of antidepressant use with risk of dementia based on propensity score matching (PSM).

|  | Non-antidepressant use | Antidepressant use | *p* |
| --- | --- | --- | --- |
| N | 33721 | 33721 |  |
| All-cause dementia |  |  |  |
| Number (%) of dementia event | 776 (2.3) | 1018 (3.0) |  |
| HR (95% CI) | Ref | **1.41 [1.28, 1.55]** | **<0.001** |
| Alzheimer’s disease |  |  |  |
| Number (%) of Alzheimer’s disease event | 336 (1.0) | 457 (1.4) |  |
| HR (95% CI) | Ref | **1.47 [1.28, 1.70]** | **<0.001** |
| Vascular dementia |  |  |  |
| Number (%) of vascular dementia event | 228 (0.7) | 275 (0.8) |  |
| HR (95% CI) | Ref | **1.31 [1.10, 1.57]** | **0.003** |

**Note:** Bolded values: *p*<0.05;

Model was adjusted for age, sex, ethnicity, socioeconomic status, less education, living alone status, excessive alcohol consumption, current smoking status, physical inactivity, sleeping duration categories, social isolation, overall health, hyperlipidemia, diabetes, hypertension, traumatic brain injury, hearing loss, vision loss, obesity, depression, PHQ-2 total score, anxiety, self-reported chronic pain, self-reported insomnia, and using other anticholinergics.

# Supplementary Table 19. Sensitivity analysis for association of antidepressant use with risk of dementia based on inverse probability of treatment weighting (IPTW).

|  | Non-antidepressant use | Antidepressant use | *p* |
| --- | --- | --- | --- |
| N | 427743 | 33721 |  |
| All-cause dementia |  |  |  |
| Number (%) of dementia event | 6904 (1.6) | 1018 (3.0) |  |
| HR (95% CI) | Ref | **1.63 [1.47, 1.80]** | **<0.001** |
| Alzheimer’s disease |  |  |  |
| Number (%) of Alzheimer’s disease event | 3320 (0.8) | 457 (1.4) |  |
| HR (95% CI) | Ref | **1.58 [1.37, 1.83]** | **<0.001** |
| Vascular dementia |  |  |  |
| Number (%) of vascular dementia event | 1584 (0.4) | 275 (0.8) |  |
| HR (95% CI) | Ref | **1.66 [1.37, 2.03]** | **<0.001** |

**Note:** Bolded values: *p*<0.05;

Model was weighted by IPTW and adjusted for age, sex, ethnicity, socioeconomic status, less education, living alone status, excessive alcohol consumption, current smoking status, physical inactivity, sleeping duration categories, social isolation, overall health, hyperlipidemia, diabetes, hypertension, traumatic brain injury, hearing loss, vision loss, obesity, depression, PHQ-2 total score, anxiety, self-reported chronic pain, self-reported insomnia, and using other anticholinergics.
